# Rationally engineering santalene synthase to readjust the component ratio of sandalwood oil

**DOI:** 10.1038/s41467-022-30294-8

**Published:** 2022-05-06

**Authors:** Wenlong Zha, Fan Zhang, Jiaqi Shao, Xingmei Ma, Jianxun Zhu, Pinghua Sun, Ruibo Wu, Jiachen Zi

**Affiliations:** 1 College of Pharmacy, Jinan University, 510632 Guangzhou, China; 2 Guangdong Provincial Key Laboratory of New Drug Design and Evaluation, School of Pharmaceutical Sciences, Sun Yat-sen University, 510006 Guangzhou, China; 3 State Key Laboratory of Bioactive Substance and Function of Natural Medicines, Institute of Materia Medica, Chinese Academy of Medical Sciences & Peking Union Medical College, 100050 Beijing, China; 4 Guangdong Provincial Key Laboratory of Traditional Chinese Medicine Informatization, 510632 Guangzhou, China

**Keywords:** Metabolic engineering, Biocatalysis, Applied microbiology

## Abstract

Plant essential oils (PEOs) are widely used in cosmetic and nutraceutical industries. The component ratios of PEOs determine their qualities. Controlling the component ratios is challenging in construction of PEO biotechnological platforms. Here, we explore the catalytic reaction pathways of both product-promiscuous and product-specific santalene synthases (i.e., SaSSy and SanSyn) by multiscale simulations. F441 of SanSyn is found as a key residue restricting the conformational dynamics of the intermediates, and thereby the direct deprotonation by the general base T298 dominantly produce *α*-santalene. The subsequent mutagenesis of this plastic residue leads to generation of a mutant enzyme SanSyn^F441V^ which can produce both *α*- and *β*-santalenes. Through metabolic engineering efforts, the santalene/santalol titer reaches 704.2 mg/L and the component ratio well matches the ISO 3518:2002 standard. This study represents a paradigm of constructing biotechnological platforms of PEOs with desirable component ratios by the combination of metabolic and enzymatic engineering.

## Abstract (web summary)

Controlling the component ratios of plant essential oils is challenging in their heterologous bioproduction. Here, the authors combine metabolic and enzymatic engineering strategies to achieve the production of sandalwood oil with a desirable component ratio in baker’s yeast.

## Introduction

**Fig. 1:**
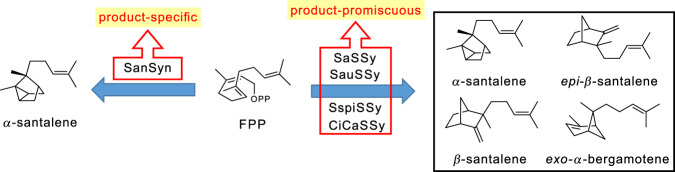
The product-promiscuous and product-specific santalene synthases. SaSSy, SauSSy, SspiSSy, and CiCaSSy produce the multiple santalene products, and SanSyn predominantly produce *α*-santalene.

Plant essential oils (PEOs) constitute a substantial and important part of commercial odorants^1^. Their ingredients have been extensively studied, and many of them are comprised of sesquiterpenes^2^, e.g., sandalwood oil. In addition to being one of the world’s most highly prized natural perfumes^1,2^, sandalwood oil has been in clinical trials for the treatment of skin disorders^3^, and also possesses other bioactivities, such as anticancer^4,5^, antihyperglycemic^6^, antioxidant^6^, and neuroprotection activities^7^. The major components of sandalwood oil are santalenes (mainly including *α*-santalene, *β*-santalene, *epi*-*β*-santalene, and *exo*-*α*-bergamotene) and santalols (mainly including *Z*-*α*-santalol, *Z*-*β*-santalol, *Z*-*exo*-*α*-bergamotol, and *Z*-*epi*-*β*-santalol) (Supplementary Fig. 1)^8^. Among these compounds, *Z*-*α*-santalol and *Z*-*β*-santalol are the most critical components contributing to the bioactivities and fragrance of sandalwood oil^5,7,9,10^. The International Organization for Standardization has issued the standard (ISO 3518:2002) for *Santalum album* L. oil in which the contents of *Z*-*α*-santalol and *Z*-*β*-santalol must fall in the 41−55% and 16−24% range, respectively^11^. Although the oil can be extracted from several *Santalum* species, the most renowned is Indian *S. album* oil. Due to overexploitation, Indian *S. album* trees are highly endangered and have been listed as Vulnerable by the International Union for the Conservation of Nature. Heterologous biosynthesis in engineered microbial hosts is a promising alternative for manufacturing plant natural products on a large scale^1,12^. In *S. album*, the biosynthetic pathway of santalols has been decoded, harboring a santalene synthase (SaSSy) and ten cytochrome P450 enzymes (CYPs) responsible for oxidizing santalenes to santalols (Supplementary Fig. 1)^13–15^. Besides, SauSSy from *S*. *austrocaledonicum*^13^, SspiSSy from *S. spicatum*^13^ and CiCaSSy from *Cinnamomum camphora*^16^ were characterized to possess the product distributions similar to that of SaSSy, and SanSyn from *Clausena lansium* was found to be a product-specific enzyme, with *α*-santalene as its predominant product (Fig. 1)^17^_._ The metabolic engineering efforts have led to construction of *α*-santalene-producing microbial platforms^18–21^. Recently, we built an engineered *Saccharomyces cerevisiae* (yeast) which can produce both santalenes and santalols, with 35.7% *Z*-*α*-santalol and 17.8% *Z*-*β*-santalol which is very close to the standard of ISO 3518:2002^22^.

As mentioned above, the component ratios of PEOs determine their qualities. Properly controlling the component ratios is challenging in construction of the heterologous biosynthetic systems of PEOs. Enzyme engineering has been proven to be a powerful approach to optimize or alternate enzymatic properties such as efficiency, stability, substrate selectivity and product profile^23^. Understanding catalytic mechanisms of enzymes is commonly believed to be the fundamental basis for redesigning the desired function. In the previous studies, we explored the mechanism which leads to catalytic promiscuity and fidelity of two sesquiterpene synthases TEAS (*Nicotiana tabacum* 5-*epi*-aristolochene synthase) and ATAS (*Aspergillus terreus* aristolochene synthase) by the combination of quantum mechanics/molecular mechanics (QM/MM) and molecular dynamics (MD)^24–27^. In this study, owing to such approach, the reaction energy profiles of both SaSSy and SanSyn are mapped out, and the interactions between the enzymes and the carbocation intermediates are explored, which lead to identification of a residue F441 in SanSyn imbuing a unique structural plasticity. And the subsequent site-saturation mutagenesis leads to generation of a mutant SanSyn^F441V^ which produces 57.2% *α*-santalene, 28.6% *β*-santalene, 6.7% *epi*-*β*-santalene and 7.6% *exo*-*α*-bergamotene, representing a desirable product profile. Meanwhile, a yeast chassis strain SZ16 (Supplementary Data 1) is obtained by optimization of the mevalonate (MVA) pathway and acetyl-CoA synthesis, knockout of the genes encoding the enzymes involved in both farnesyl diphosphate (FPP) consumption and *Z*-*α*-santalol transformation, and deletion of the yeast transcriptional repressor ROX1. Then, *SanSyn*^*F441V*^ is used to construct the santalene/santalol-producing yeast from SZ16 (Supplementary Data 1). The resulting SZ24 (Supplementary Data 1) totally produces 704.2 mg/L santalenes and santalols, with 43.4% *Z*-*α*-santalol, 22% *Z*-*β*-santalol and only 6.7% *Z*-*exo*-*α*-bergamotol which well matches the standard of ISO 3518:2002.

## Results

### QM/MM simulations of SaSSy and SanSyn catalysis

CYP736A167 exhibits the approximately equal substrate preference to *α*-santalene, *β*-santalene, *epi*-*β*-santalene and *exo*-*α*-bergamotene, and hence the component profile of sandalwood oil is shaped mainly by SaSSy^22^. As mentioned above, *Z*-*α*-santalol and *Z*-*β*-santalol are the most important components which contribute to the odor and bioactivity of sandalwood oil, and used for quality control in ISO 3518:2002. However, SaSSy produces much *exo*-*α*-bergamotene, resulting in the large ratios of *exo*-*α*-bergamotene and *Z*-*exo*-*α*-bergamotol in the fermentation oil, which are close to those of *α*-santalene and *Z*-*α*-santalol and even larger than those of *β*-santalene and *Z*-*β*-santalol^22^. In this study, we sought to elevate the ratios of *Z*-*α*-santalol and *Z*-*β*-santalol through engineering santalene synthase to attenuate the production of *exo*-*α*-bergamotene and *Z*-*exo*-*α*-bergamotol. To this end, we first explored the catalytic mechanisms of SaSSy and SanSyn by QM/MM calculation.

**Fig. 2:**
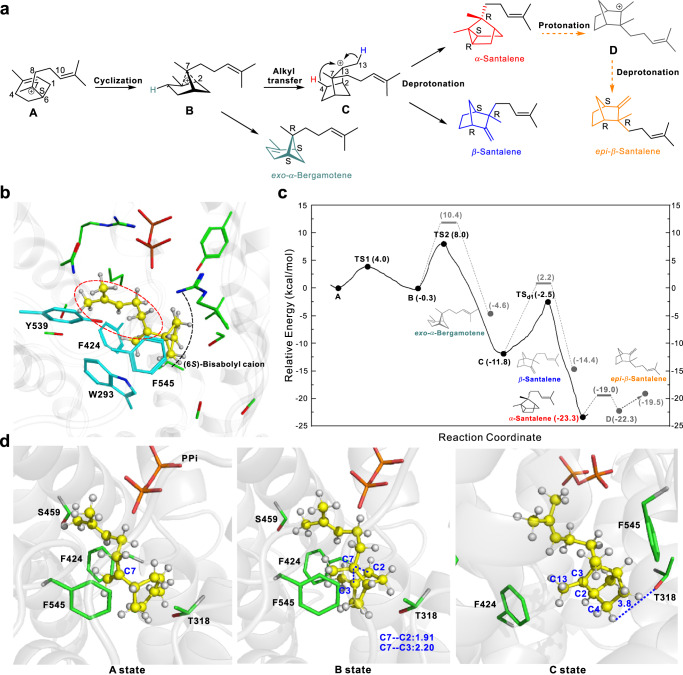
QM/MM MD simulation of the reaction pathway catalyzed by SaSSy. **a** The reaction pathway from (6*S*)-bisabolyl cation (A state) to the multiple products. **b** A state in the active site of SaSSy model. **c** Relative energy profiles from A state to *α*-santalene. **d** Representative structures of QM/MM simulations for A, B, and C states are shown.

**Fig. 3:**
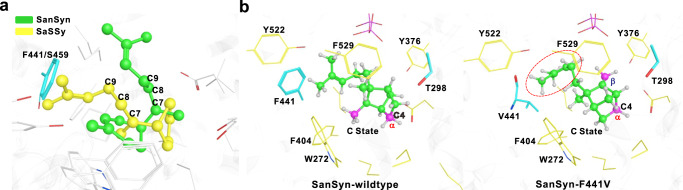
Comparative analysis of the active cavities of SaSSy, SanSyn, and SanSyn^F441V^. **a** Comparisons of the A state conformations in SaSSy (yellow) and SanSyn (green). **b** The C state conformations in SanSyn and SanSyn^F441V^. The space released by F441V mutation is indicated by the red dashed line.

SaSSy produces multiple bridged ring products, as shown in Fig. 1. Among these compounds, *α*-santalene and *β*-santalene could be directly derived from (6*S*)-bisabolyl cation (A state) through cyclization, alkyl transfer and deprotonation after initial 1,6-closure of farnesyl diphosphate, while deprotonation from B state leads to *exo*-*α*-bergamotene (Fig. 2a). To date, the structures of both holo-SaSSy and holo-SanSyn are still absent, although the apo-SaSSy structure (PDB ID: 5ZZJ) has been reported^28^. Thus, the SaSSy and SanSyn models were predicted using Alphafold2 herein (Supplementary Fig. 2)^29^, and A state was considered in the active sites (see Figs. 2 and 3 and Supplementary Method 1). As shown in Fig. 2b, the conformation of A state is preorganized for further cyclization with the homoprenyl group surrounded by the aromatic residues (e.g., F545 and F424) and the cyclohexene ring on the other side of the pocket in SaSSy. The 7,2(3)-cyclization of A state conquers 4.0 kcal/mol barrier to yield a non-classical carbocation, namely B state (Fig. 2a, c). The subsequent alkyl transfer leads to C state with notable heat release (Fig. 2a, c). As shown in Fig. 2d, B state is stabilized by the aromatic residues (F424 and F545), and the subsequent alkyl transfer from B to C state has to overcome a barrier of 8.3 kcal/mol (Fig. 2c), indicating that B state has a lifetime to a certain extent and the intrinsic conformational dynamics allow it to produce *exo*-*α*-bergamotene. After F424 and F545 were respectively replaced by alanine, the catalytic efficiency of two mutants SaSSy^F545A^ and SaSSy^F424A^ dramatically decreased (Supplementary Figs. 3a, b and 4a, b), supporting their substantial contribution to configuring of the intermediate conformation. And we found that T318 in SaSSy might appropriately serve as the major general base for deprotonation at C-4 (Fig. 2d) to yield *α*-santalene across a barrier of 9.3 kcal/mol (Fig. 2c) which indicates that the deep energy well of C state confers a considerable lifetime to it, relatively longer than B state. Besides, the unoccupied space of the active pocket is enlarged as the bridged ring formation at C state (Fig. 2d), further increasing the conformational dynamics of the carbocation intermediate at C state. As a result, deprotonation from C-13 to yield *β*-santalene is also feasible by the help of T318 (Fig. 2a, d). This was supported by the T318A mutation which resulted in almost complete loss of the ability to produce *α*-santalene and *β*-santalene (Supplementary Fig. 3c and 4c). Regarding to the minor product *epi*-*β*-santalene, it could be directly derived only from (6*R*)-bisabolyl cation but not A state, i.e., (6*S*)-bisabolyl cation, if going through the reaction pathway similar to synthesis of *β*-santalene (Fig. 2a and Supplementary Fig. 5). However, it was found that the (6*R*)-bisabolyl cation conformation is not likely existed in SaSSy, since none of the corresponding reasonable conformations could be well maintained in QM/MM MD simulations. In addition, the other compounds (such as 7-*epi*-*α*-santalene) possibly derived from the *R*-configurational pathway are absent in the product mixture of SaSSy (Supplementary Fig. 5), which further suggests the absence of (6*R*)-bisabolyl cation in the reaction pathway catalyzed by SaSSy. Alternatively, it was reported that *epi*-*β*-santalene could derive from cleavage of the cyclopropane ring of *α*-santalene (Supplementary Fig. 6)^30^. Our calculation proved that the cyclopropane ring opening of *α*-santalene is feasible (only 4.3 kcal/mol barrier) (Fig. 2c), which could be triggered through attracting a proton from the T318. This leads to generation of D state, and the subsequent deprotonation from D state results in the production of *epi*-*α*-santalene (Supplementary Figs. 6 and 7).

**Fig. 4:**
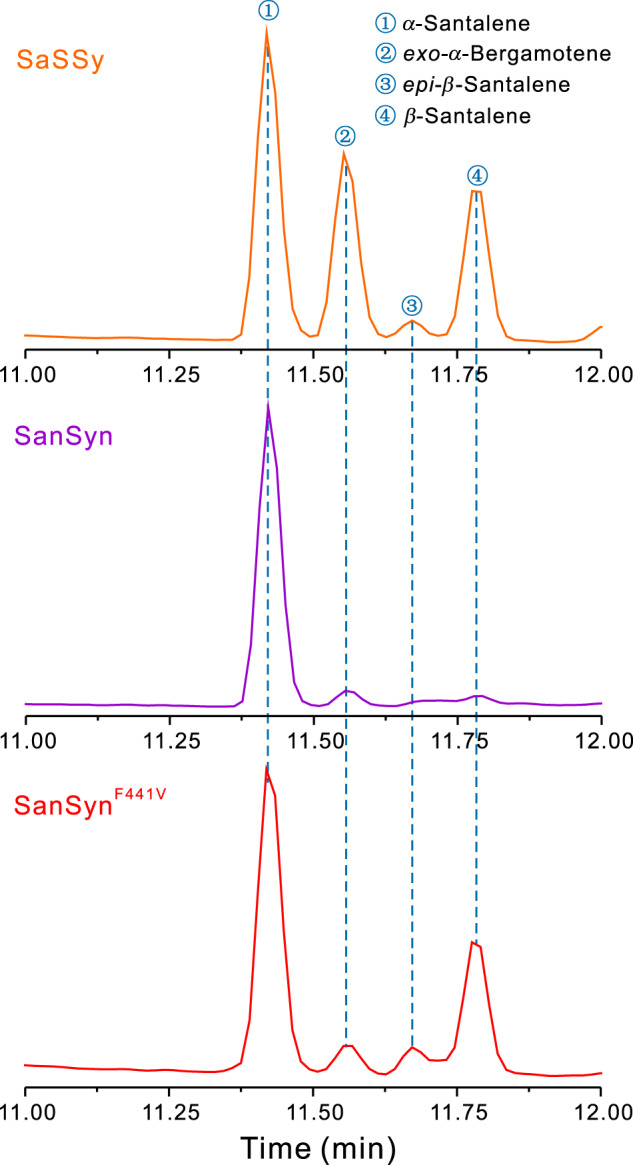
GC-MS analysis of the products of SaSSy, SanSyn and SanSyn^F441V^. SanSyn^F441V^ can produce both *α*-santalene and *β*-santalene.

Compared with SaSSy, SanSyn dominantly produces *α*-santalene, with only a trace of the other relevant products (Fig. 4), suggesting that the conversion from B state to C state and the followed H4 leaving to the C3-C4 bond formation is dominant. As shown in Fig. 3a, the conformation of A state in SanSyn is more compact than that in SaSSy, which means the unoccupied space for the intermediate conformational dynamics is limited in SanSyn. The homoprenyl group of A state is well oriented for hyperconjugation as an aligned conformer to stabilize the carbocation in SanSyn, while more flexible as an extended conformer in SaSSy. The effect of hyperconjugation donation on the relative stability of B state has been previously discussed by the extensive QM calculations^31^. Herein the QM/MM calculations from A to C states in SanSyn (Supplementary Fig. 8) validated such hyperconjugation effect that the potential energy well of B state in SanSyn (depth of 2.2 kcal/mol) is shallower than that in SaSSy (depth of 4.3 kcal/mol), indicating a shorter lifetime of B state in SanSyn. Meanwhile, the limited unoccupied space configures the compact conformation of B state preferable to the conversion to C state, thus largely avoiding the production of *exo*-*α*-bergamotene in SanSyn. Subsequently, T298 corresponding to T318 in SaSSy (Supplementary Fig. 9) could serve as the potential major general base to achieve deprotonation at C-4 for the production of *α*-santalene. This was supported by the mutagenesis experiment in which the replacement of T298 with alanine resulted in a significant decrease in the catalytic efficiency (Supplementary Figs. 3i and 4e). Comparing Sansyn and SaSSy, we found that the lack of unoccupied space in Sansyn would largely restrict the intermediate conformational dynamics, which is also a key factor for its high fidelity. We, therefore, surmised that the replacement of the residue containing a large side chain with a proper small one in the cavity to vacate more space for the intermediate (especially C state) conformational dynamics might achieve our end. Based on the structure comparisons between SanSyn and SaSSy and our understanding of the reaction pathways, a single amino acid variant F441/S459 (SanSyn in front) attracted our attention (Fig. 3a). In SanSyn, F441 appears to restrict the conformational space of the intermediates (Fig. 3b), thereby likely playing a critical role in guiding the intermediate conformation.

### Creation of SanSyn^F441V^ with a desirable product profile

Given that F441 of SanSyn corresponds to S459 of SaSSy, we conducted site-directed mutagenesis to obtain SanSyn^F441S^. The yeast strain WL07^22^ expressing SanSyn^F441S^ produced both *α*-santalene and *β*-santalene (Supplementary Fig. 3d), which encouraged us to conduct site-saturation mutagenesis of F441. The results showed that the replacement of F441 with the residues (including threonine, valine, leucine and isoleucine) similar to serine resulted in the production of both *α*-santalene and *β*-santalene (Fig. 4 and Supplementary Figs. 3e−g and 4d). Among these mutants, SanSyn^F441V^ exhibited the highest efficiency and a desirable product profile, 57.2% *α*-santalene, 28.6% *β*-santalene, 6.7% *epi*-*β*-santalene and 7.6% *exo*-*α*-bergamotene (Fig. 4). QM/MM MD simulations of C state in SanSyn and SanSyn^F441V^ were performed. As shown in Fig. 3b, C state adopts a compact conformation in SanSyn. While in SanSyn^F441V^, the homoprenyl group occupies the additional space arising from F441V mutation and adopts a more extended conformation. As observed in QM/MM MD and traditional MD simulations, the conformational flexibility of C state in SanSyn^F441V^ is more notable with larger fluctuations of the dihedral C6–C7–C8–C9 (Supplementary Figs. 10 and 11) compared to that in the wildtype SanSyn, which brings about the rotatability of the bridged ring moiety and thus deprotonation could occur at more than one position (i.e., C-4 and C-13). Besides, the long lifetime of C state would further enhance the conformational dynamics of the carbocation intermediate at this state. As a result, T298 could eliminate both H4 and H13 of C state for the production of *α*-santalene and *β*-santalene, respectively, as we surmised above (Fig. 3b and Supplementary Fig. 12). Indeed, the replacement of T298 with alanine in SanSyn^F441V^ led to substantial loss of the ability to produce *α*-santalene and *β*-santalene (Supplementary Figs. 3h and 4f). These results document that the structural features, especially the cavity space, substantially contribute to configure preorganized substrate folding modes and give rise to various intermediate conformations with different dynamic behavior^25,32^.

### Enhancement of the santalene and santalol titers by metabolic engineering

**Fig. 5:**
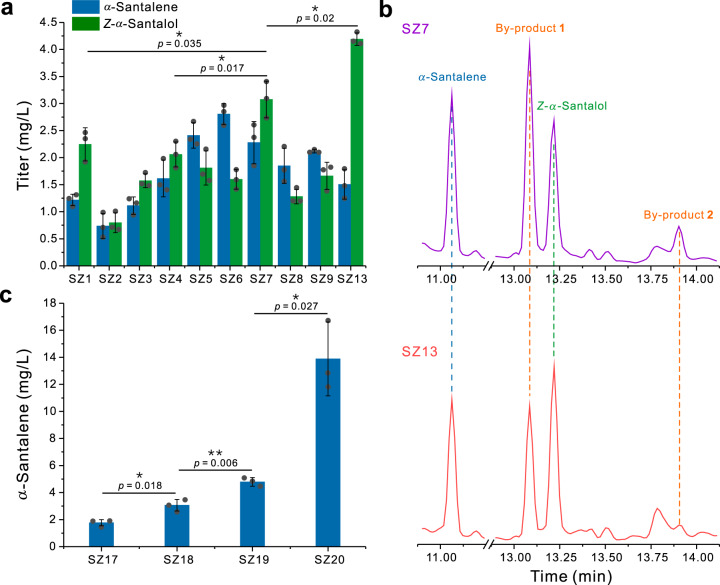
Metabolic engineering of yeast for the production of santalenes and santalols. **a** The production of *α*-santalene and *Z*-*α*-santalol by expression of SanSyn and CYP736A167 assisted with the different CPRs in the yeast hosts (SZ1-SZ9, see Supplementary Data 1), and enhanced the production of *α*-santalene and *Z*-*α*-santalol by knockout of OYE2, OYE3, ATF1, and ATF2 (SZ13, see Supplementary Data 1). **b** GC-MS analysis of *α*-santalene, *Z*-*α*-santalol, dihydro-*α*-santalol (by-products 1) and acetyl dihydro-*α*-santalol (by-product 2) before and after knockout of OYE2, OYE3, ATF1, and ATF2 (SZ7 and SZ13, see Supplementary Data 1). **c** Enhanced the production of *α*-santalene by overexpression of IDI1, UPC2-1, ADH2, ALD6, and ACS^L641P^, and knockdown of ERG9 (SZ18−SZ20, see Supplementary Data 1). All data represent the mean of *n* = 3 biologically independent samples and error bars show standard deviation. Statistical analysis was performed by using two-tailed and two-sample unequal variance *t* test (*P < 0.05, **P < 0.01). Source data are provided as a Source Data file.

In order to easily quantify the titers, the product-specific enzyme SanSyn was utilized in this part. We put forth the first effort to screen the initial yeast host strains including *S. cerevisiae* BY4741, CEN.PK2-1C and CEN.PK2-1D (Supplementary Data 1). These three strains were transformed with pSZ1 for expressing tHMG1 (the truncated 3-hydroxy-3-methylglutaryl coenzyme A reductase, a limited enzyme in terpene synthesis)^33^ and SanSyn and pSZ2 for expressing CYP736A167 and SaCPR2 (a cytochrome P450 reductase from *S. album*) (Supplementary Data 2 and 3)^22^. The highest total *α*-santalene and *Z*-*α*-santalol titer (5.4 mg/L) was observed when using *S. cerevisiae* CEN.PK2-1D (SZ7, Supplementary Data 1), ~1.5-folds relative to those when using *S. cerevisiae* BY4741 (SZ1, Supplementary Data 1) and CEN.PK2-1C (SZ4, Supplementary Data 1), respectively (Fig. 5a). Because cytochrome P450 reductases play important roles in the functions of cytochrome P450 enzymes and significantly influence their efficiencies^34,35^, we tested the efficiency of CYP736A167 assisted by two other cytochrome P450 reductases ATR1 (from *Arabidopsis thaliana*)^36^ and CrCPR (from *Catharanthus roseus*)^37^, respectively (Supplementary Data 1 and 2). However, the better efficiency was not achieved (Fig. 5a). Thus, SaCPR2 was still used in the following modification.

In addition to *α*-santalene and *Z*-*α*-santalol, two unknown peaks were detected by GC-MS from the culture of SZ7 (Fig. 5b). The titer of by-product 1 was 4.3 mg/L which was even higher than the *Z*-*α*-santalol titer (Fig. 5b). As the MS spectral profiles of these two by-products are very similar to that of *Z*-*α*-santalol (Supplementary Fig. 13), we surmised that they derived from *Z*-*α*-santalol through the reactions catalyzed by yeast endogenous enzymes. From 12 L SZ7 culture, these two by-products were isolated and their structures were respectively determined as dihydro-*α*-santalol and acetyl dihydro-*α*-santalol by NMR analysis (Supplementary Figs. 14–17)^38^. It has been reported that OYE2 and OYE3 (two yeast old yellow enzymes) can reduce geraniol into citronellol^39–41^ and ATF1 and ATF2 (two yeast alcohol acetyltransferases) can catalyze acetylation of geraniol^39–41^. We assumed that these four endogenous enzymes may also play roles in derivatization of *Z*-*α*-santalol. Indeed, knockout of OYE2 and OYE3 by CRISPR/Cas9 (SZ12, Supplementary Data 1) resulted in a 27.9% decrease of the dihydro-*α*-santalol titer to 3.1 mg/L and a 23.1% increase of the *Z*-*α*-santalol titer to 3.8 mg/L compared with those of SZ7 (Fig. 5a and Supplementary Fig. 18). This indicates the presence of the other endogenous enzymes which can catalyze hydrogenation of *Z*-*α*-santalol to dihydro-*α*-santalol. Then, all of OYE2, OYE3, ATF1, and ATF2 in *S. cerevisiae* CEN.PK2-1D were knocked out to afford SZ11 (Supplementary Data 1). After transforming SZ11 with pSZ1 and pSZ2, the *Z*-*α*-santalol titer in the culture of the resulting strain SZ13 was 4.2 mg/L, a 35.5% increase relative to that of SZ7 (Fig. 5a), and the by-product 2 acetyl dihydro-*α*-santalol was undetectable (Fig. 5b).

Subsequently, the terpene biosynthetic pathways in yeast were engineered. Only SanSyn was expressed in the engineered strains in this part to simplify the experimental process, and thus *α*-santalene served as the sole reference compound for weighing the effect of each modification. The mevalonate (MVA) pathway was firstly optimized by integration of one copy of *IDI1* encoding isopentenyl diphosphate isomerase^42^ and *UPC2-1* encoding a mutant of the transcription factor UPC2 which can enhance expression of the genes of yeast MVA pathway^22^. Because DPP1 (a yeast diacylglycerol pyrophosphate phosphatase) can dephosphorylate FPP into farnesol^18^, we integrated one copy of *IDI1* and *UPC2-1* into *DPP1* locus for both optimization of MVA pathway and attenuation of FPP consumption through dephosphorylation. After transformation with pSZ5 harboring *SanSyn* and *tHMG1* (Supplementary Data 2 and 3), the resulting SZ18 produced 3.1 mg/L *α*-santalene, a 73% increase relative to that of SZ17 generated by introducing pSZ5 into SZ11 (Fig. 5c and Supplementary Data 1). In order to increase the supply of acetyl-CoA (the precursor of MVA pathway) and further decrease FPP consumption through dephosphorylation, we enhanced conversion from ethanol to acetyl-CoA by integrating ADH2 (a yeast alcohol dehydrogenase), ALD6 (a yeast acetaldehyde dehydrogenase) and ACS^L641P^ (the L641P variant of a *Salmonella enterica* acetyl-CoA synthetase)^20^ into the locus of *LPP1*, encoding a yeast lipid phosphate phosphatase which, like DPP1, can also dephosphorylate FPP^18^. The *α*-santalene titer of the resulting stain SZ19 (Supplementary Data 1) increased to 4.8 mg/L (Fig. 5c). To further redirect more FPP to *α*-santalene biosynthesis, ERG9 (yeast squalene synthase) was downregulated to attenuate FPP consumption in triterpenoid/steroid biosynthesis by replacing its native promotor with a glucose-induced promotor P_*HXT1*_, which led to an *α*-santalene titer of 13.9 mg/L in the resulting SZ20 (Fig. 5c). Thus, the corresponding chassis strain SZ16 (Supplementary Data 1) with the optimized MVA and acetyl-CoA synthesis pathways and *ERG9* knockdown was used to construct the santalene/santalol-producing strains.

**Fig. 6:**
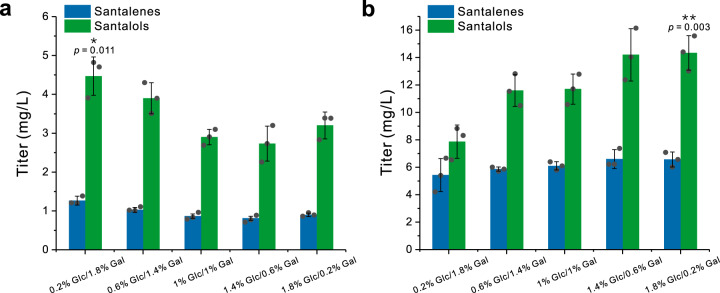
The titers of santalenes and santalols in SZ21 and SZ22. The titers of santalenes and santalols in SZ21 (with two copies of *SanSyn*^*F441V*^ and one copy of *tHMG1* and *CYP736A167*) (**a**) and SZ22 (with three copies of *SanSyn*^*F441V*^ and two copies of *tHMG1* and *CYP736A167*) (**b**) when fermented with the different ratios of glucose and galactose. All data represent the mean of *n* = 3 biologically independent samples and error bars show the standard deviation. Statistical analysis was performed by using two-tailed and two-sample unequal variance *t* test (*P < 0.05, **P < 0.01). Source data are provided as a Source Data file.

Because expression of biosynthetic genes by the integration mode often give rise to higher and more stable yields of the corresponding compounds^43^, we integrated one copy of *tHMG1*, *CYP736A167* and *SaCPR2* and two copies of *SanSyn*^*F441V*^ into SZ16 to afford SZ21 (Supplementary Data 1). In SZ21 culture, the titers of santalenes (*α*-santalene, *β*-santalene, *epi*-*β*-santalene and *exo*-*α*-bergamotene) and santalols (*Z*-*α*-santalol, *Z*-*β*-santalol, *Z*-*exo*-*α*-bergamotol, and *Z*-*epi*-*β*-santalol) were 1.3 mg/L and 4.5 mg/L, respectively (Fig. 6a), when fermented under the optimal condition with 0.2% w/w glucose and 1.8% w/w galactose. The previous studies reported that deletion of *ROX1* (a yeast transcriptional repressor) elevated the expression levels of terpenoid and steroid biosynthetic genes^43,44^. Thus, we integrated an additional copy of *tHMG1*, *SanSyn*^*F441V*^ and *CYP736A167* into *ROX1* locus of SZ21 to obtain SZ22 (Supplementary Data 1). SZ22 produced 6.6 mg/L santalenes and 14.3 mg/L santalols in the optimal condition, respectively corresponding to 5.1- and 3.2-fold increase relative to those of SZ21 (Fig. 6b). The ratios of *Z*-*α*-santalol and *Z*-*β*-santalol in the santalene and santalol mixture produced by SZ22 were 48.1% and 20.2%, respectively, which well matches the ISO 3518:2002 standard.

### Fed-batch fermentation and tuning of the copy numbers of *SanSyn*^*F441V*^ and *CYP736A167*

**Fig. 7:**
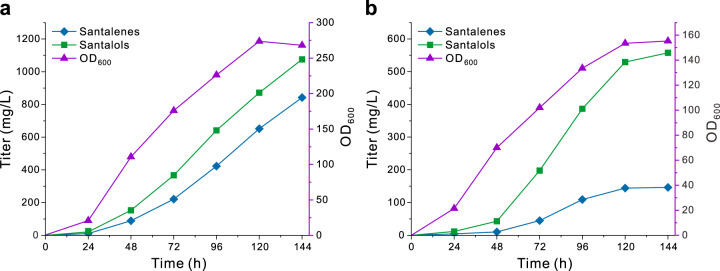
Fed-batch fermentation of SZ22 (with three copies of *SanSyn*^*F441V*^ and two copies of *CYP736A167*) and SZ24 (with three copies of *SanSyn*^*F441V*^ and *CYP736A167*). The santalenes and santalols titers and OD_600_ of SZ22 (**a**) and SZ24 (**b**). All data represent the mean of *n* = 2 biologically independent samples. Source data are provided as a Source Data file.

Because galactose is too expensive for industry-scale fermentation, we sought to use the cheaper carbon source. Given that the route from ethanol to acetyl-CoA in SZ22 was optimized, the mixture of glucose and ethanol was utilized in the fed-batch fermentation. In the batch media, glucose (25 g/L) was the sole carbon source. When glucose was completely consumed, the significant OD increase was observed. And at this point the feeding phase started. To optimize the feeding media, three different ratios of glucose and ethanol were tested, including 350 g/L glucose and 150 g/L ethanol (Supplementary Fig. 19a), 250 g/L glucose and 250 g/L ethanol (Supplementary Fig. 19b), and 150 g/L glucose and 350 g/L ethanol (Fig. 7a). The highest titer (842.7 mg/L santalenes and 1075.7 mg/L santalols) was observed when 150 g/L glucose and 350 g/L ethanol were used (Fig. 7a). The ratios of *Z*-*α*-santalol and *Z*-*β*-santalol were 29.4% and 15% (Table 1), respectively, indicating that much santalenes were not oxidized into santalols. Therefore, we increased the copy number of *CYP736A167* to yield SZ23 and SZ24 (Fig. 7b, Supplementary Fig. 20, and Supplementary Data 1). 43.4% *Z*-*α*-santalol and 22.0% *Z*-*β*-santalol were observed in the oil produced by SZ24 (Table 1) which well matches the ISO 3518:2002 standard, but the total titer of santalenes and santalols decreased to 704.2 mg/L (Fig. 7b). The titer decrease might result from the biomass decrease, as the OD of SZ24 was approximately 58% of that of SZ22 (Fig. 7). This observation indicated that the high copy number of *CYP736A167* could inhibit yeast growth. In the next research, CYP736A167 should be also engineered to increase it catalytic efficiency, and hence the higher conversation of santalenes to santalols would be achieved using the low copy number of the resulting mutant. In addition, intriguing was that the ratios between dihydro-*α*-santalol and *Z*-*α*-santalol in the oils produced by SZ22 and SZ24 were only 1:17 and 1:20, respectively (Supplementary Fig. 21). This indicates that the catalytic capacity of the yeast endogenous enzymes responsible for hydrogenation of *Z*-*α*-santalol is limited, and thereby the ratio between dihydro-*α*-santalol and *α*-santalol significantly decreased when the *Z*-*α*-santalol titer was dramatically elevated.

**Table 1:** The component ratios of the oils produced by SZ22 and SZ24.

Component	SZ22	SZ24
*Z*-*α*-Santalol	29.4%	43.4%
*Z*-*β*-Santalol	15.0%	22.0%
*Z*-*exo*-*α*-Bergamotol	6.5%	6.7%
*Z*-*epi*-*β*-Santalol	5.2%	7.1%
*α*-Santalene	20.8%	9.8%
*β*-Santalene	12.6%	5.8%
*exo*-*α*-Bergamotene	5.1%	2.0%
*epi*-*β*-Santalene	5.4%	3.2%

Source data are provided as a Source Data file.

## Discussion

The qualities of plant natural product mixtures (including PEOs, herbal medicines, etc.) are determined by their component ratios. Accordingly, to accomplish biotechnological production of the high-quality products requires not only enhancement of yields not also optimization of component ratios. In the biosynthetic pathways of these compound mixtures, some product-promiscuous enzymes often play critical roles in shaping their component ratios. Clarification of catalytic mechanism of these enzymes would enable optimization of product distribution by enzymatic engineering^45,46^. In sandalwood oil biosynthesis, SaSSy produces a large amount of *exo*-*α*-bergamotene, which leads to the ratio of *Z*-*exo*-*α*-bergamotol comparable to those of *Z*-*α*-santalol and *Z*-*β*-santalol (two most valuable components) in the oil produced by the engineered yeast constructed with SaSSy^22^. In this study, we established the models of SaSSy and SanSyn using Alphafold2, and simulated their catalytic reaction pathways using QM/MM calculation.

In SaSSy model, only (6*S*)-bisabolyl cation (A state) (Fig. 2) is likely existed, while (6*R*)-bisabolyl cation isn’t. And the possible products derived from (6*R*)-bisabolyl cation are absent. These results indicate that the substrate folding mode in the cavity is well preorganized, which determines the fate of the reaction pathway. It has been reported that aromatic amino acid residues in terpene cyclases can stabilize carbocation intermediates through cation−π interactions^47^. Indeed, mutagenesis of F545 and F424 resulted in a significant loss of catalytic efficiency (Supplementary Figs. 3a, b and 4a, b).

Comparative analysis of SaSSy and SanSyn showed they respectively harbor a bigger and a smaller cavities (Fig. 3b). Accordingly, the intermediates in SaSSy adopt the flexible conformations with different dynamic behavior, which allow a versatile reaction pathway for the production of multiple products (Fig. 1). Oppositely, the limited cavity space in SanSyn confers the compact conformations to the intermediates, which makes it a product-specific enzyme. Therefore, the residues highly related to the cavity space may substantially influence the conformation dynamic of the intermediates, and thereby mutagenesis of these residues could alter the product distribution. F441 in SanSyn was considered to be the residue of this type base on QM/MM MD simulations (Fig. 3). The additional space released by F441V mutation was observed (Fig. 3b), and the resulting SanSyn^F441V^ exhibited a desirable product profile, 57.2% *α*-santalene, 28.6% *β*-santalene, 6.7% *epi*-*β*-santalene and 7.6% *exo*-*α*-bergamotene. Meanwhile, metabolic engineering was performed, which led to the establishment of the chassis strain SZ16 by optimization of MVA pathway and acetyl-CoA synthesis, knockout of the genes encoding the enzymes involved in both FPP consumption and *Z*-*α*-santalol transformation, and deletion of the yeast transcriptional repressor ROX1. Next, *SanSyn*^*F441V*^ was used for construction of the santalene/santalol-producing yeast strains (Supplementary Fig. 22). After tuning of the copy numbers of *SanSyn*^*F441V*^ and *CYP736A167*, SZ24 totally produced 704.2 mg/L santalenes and santalols by a fed-batch fermentation, with 43.4% *Z*-*α*-santalol and 22.0% *Z*-*β*-santalol which well matches the ISO 3518:2002 standard (Table 1).

The active side chains of the amino acids locating at the cavity of terpene cyclases often serve as general bases for deprotonation to produce olefins^27^. Herein, QM/MM MD simulations and site-directed mutagenesis suggested that T318 in SaSSy and T298 in SanSyn are the major bases for deprotonation at C-4 and C-13 of C state (Figs. 2d, 3b and Supplementary Figs. 3c, i and 4c, e). This indicates that the residues serving as the general bases in SaSSy and SanSyn are highly conserved although these two enzymes only share a 33% sequence identity (Supplementary Fig. 9).

This study documents that multiscale simulations substantially facilitate the identification of key amino acid residues associated with conformational dynamics of intermediates in key enzymes, and the resulting findings can lead to redesign of the product distribution. Such enzymatic engineering approach, in association with metabolic engineering, represents a general strategy for construction of microbial platforms of not only PEOs but also other valuable natural product mixtures.

## Methods

### Computational details

All the computational details, including system setup, classical MD simulations and QM/MM MD simulations, are included in Supplementary Method 1. The PDB files of SaSSy and SanSyn computational models including predicted models and the important states of QM/MM simulations are provided in Supplementary Data 4.

### Strains and media

The *S. cerevisiae* strains BY4741, CEN.PK2-1D, and CEN.PK2-1C were maintained on YPD plates at 30 °C. Engineered yeast strains were grown in SD media lacking corresponding amino acids. *Escherichia coli* DH10B was used for genes cloning and plasmids construction, and cultivated at 37 °C in NZY media; *E*. *coli* BL21 (DE3) was used for protein expression, and cultivated at 16 °C in LB media.

### Mutagenesis of SaSSy and SanSyn, and construction of gene expression cassettes and plasmids

The genomic DNA of *S. cerevisiae* CEN.PK2-1D was obtained using the DNAiso reagent (Takara Biomedical Technology Co., Ltd., Beijing, China.). Polymerase Chain Reactions (PCR) were performed using Phanta Max Super-Fidelity DNA Polymerase (Vazyme Biotech Co., Ltd.). The yeast endogenous genes, promotors, terminators, and homology arms were amplified from CEN.PK2-1D genomic DNA. The gene expression cassettes were prepared by overlap PCR (Supplementary Fig. 23)^48^. The plasmids were constructed using ClonExpress II One Step Cloning Kit. The resulting plasmids were confirmed by gene sequencing. To obtain the mutants of SaSSy and SanSyn, SaSSy and SanSyn were respectively ligated into the pEASY Blunt Cloning Vector (TransGen Biotech Co., Ltd.), and the resulting plasmids were used as the templates for mutagenesis by a PCR-based method^49^. Expression cassette fragments were amplified from the corresponding plasmids. All the plasmids and primers are listed in Supplementary Data 2 and 3.

### Strain construction

The expression cassette fragments were amplified from the corresponding plasmids, and subsequently integrated into the yeast chromosome. Yeast transformation was performed by Frozen-EZ Yeast Transformation II Kit (Zymo Research Co., Ltd.). All the strains are listed in Supplementary Data 1.

For CRISPR/Cas9-mediated knockout^50^, the complete crRNA arrays were synthesized by GenScript Co., Ltd (Nanjing, China), and ligated into the vector pCRCT. The resulting plasmids were introduced into the yeast strains. After growing on plates for 4 days, the colonies were inoculated into 10-mL test tube containing 5 mL SD-URA media. 100 μL of each cell culture was transferred into 5 mL fresh SD-URA media. After incubation for 6 days, the diluted cell culture was plated onto SD-URA media plate, and incubated at 30 °C for 3 days. Then, the single colonies were picked and tested by colony PCR. Finally, the pCRCT plasmids were removed by successive subculturing.

### Flask cultivation of yeast strains

The single colonies of each engineered strain were inoculated into 10-mL test tubes containing 5 mL SD media without corresponding nutrition at 30 °C and 230 rpm for 24 h. Then, 1 mL starter culture was inoculated into a 250-mL flask containing 50 mL SD media and grown at 30 °C and 230 rpm for 4 days.

### Separation and structural elucidation of by-products

In all, 12 L SZ7 culture was extracted with ethyl acetate (EtOAc). The crude extract was preliminarily purified by silica gel column chromatography, with gradually increasing EtOAc in petroleum ether (PE) as the mobile phase. Two by-products were detected by GC-MS in the fractions eluted by 30:1 and 20:1 PE/EtOAc solutions, respectively. These two fractions were further subjected to high-performance liquid chromatography (HPLC) performed on an Ultimate 3000 instrument (Thermo Scientific, USA) with a YMC-Pack Pro C18 (5 μm, 4.6 × 250 mm) column (the mobile phase: 50–100% methanol in water within 0.00–35.00 min). The structures of the by-products were determined by NMR on TopSpin 2.1 (Bruker).

### Fed-batch cultivation of yeast strains

Fed-batch fermentation was performed in a 5-liter fermentor. The agitation ranged from 250 to 800 rpm, the airflow rate ranged from 2 to 3 L/min to keep the dissolved oxygen (DO) above 40%, and the flow rate of feeding media ranged 2 to 14 mL/h. 1 L batch medium contained 15 g (NH_4_)_2_SO_4_, 8 g KH_2_PO_4_, 3 g MgSO_4_, 0.72 g ZnSO_4_.7H_2_O, 12 mL vitamin solution, 10 mL trace metals and 25 g glucose. The trace metal solution (per L) contained 15 g EDTA, 10.2 g ZnSO_4_.7H_2_O, 0.5 g MnCl_2_.4H_2_O, 0.5 g anhydrous CuSO_4_, 0.86 g CoCl_2_.6H_2_O, 0.56 g Na_2_MoO_4_.2H_2_O, 3.84 g CaCl_2_.2H_2_O, and 5.12 g FeSO_4_.7H_2_O. The vitamin solution contained (per L) contained 0.05 g biotin, 1 g calcium pantothenate, 1 g nicotinic acid, 25 g myoinositol, 1 g thiamine hydrochloride, 1 g pyridoxal hydrochloride, and 0.2 g *p*-aminobenzoic acid. The feed media (per L) contained 10 mL trace metal solution, 12 mL vitamin solution, 9 g KH_2_PO_4_, 2.5 g anhydrous MgSO_4_, 3.5 g K_2_SO_4_, 0.28 g Na_2_SO_4_, and total 500 g glucose and ethanol (with various ratios).

### Protein expression and purification

pET28a and pCold TF were respectively used to construct the plasmids for expression of the SaSSy and SanSyn mutants in *E. coli* BL21 (DE3) (Supplementary Data 2)^51^. Each transformant was grown in LB medium at 37 °C, shaking at 220 rpm, when the OD_600_ reached 0.4−0.6, 0.4 mM isopropyl *β*-d-1-thiogalactopyranoside (IPTG) was used to induce the protein expression at 16 °C for 16 h. The cells were harvested by centrifugation at 4000×*g* for 5 min at 4 °C, and resuspended in buffer D (50 mM Tris-HCl, 5 mM MgCl_2_, 250 mM NaCl, 10% glycerol, pH 7.5). The cells were lysed and centrifuged at 13,000×*g* for 10 min to obtain the supernatant crude protein.

SaSSy mutant protein was subjected to a Ni-NTA affinity column successively eluted with buffer A (50 mM Tris-HCl, 250 mM NaCl, 5 mM MgCl_2_, 10% glycerol, pH 8.4), buffer B (50 mM Tris-HCl, 250 mM NaCl, 5 mM MgCl_2_, 10% glycerol, 100 mM imidazole, pH 8.4), and buffer C (50 mM Tris-HCl, 250 mM NaCl, 5 mM MgCl_2_, 10% glycerol, 300 mM imidazole, pH 8.4). After the removal of imidazole by dialysis, the resulting enzyme solution was concentrated by centrifugation.

### In vitro enzymatic reactions

Enzymatic reactions were performed in 500 μL buffer D (50 mM Tris-HCl, 5 mM MgCl_2_, 250 mM NaCl, 10% glycerol, pH 7.5) containing 5 μM enzyme and 3 mM FPP (Sigma). The reaction mixtures were incubated at 30 °C for 3 h, followed by extraction with n-hexane for GC-MS analysis. For the SanSyn mutants, the reaction mixtures were established with the crude proteins which were prepared by centrifugation of *E. coli* (expressing the SanSyn mutants) cell lysates at 13,000 × *g* for 10 min.

### Extraction and analysis of santalols and santalenes

The cultures were extracted twice with an equal volume of EtOAc. The EtOAc layers were combined and dried in vacuum, and then the residues were dissolved in hexane for GC-MS analysis.

Fermentation products were analyzed on an Agilent GC-MS instrument (7890B/5977B) equipped with an HP-5MS column. The correlation co-efficients of *α*-santalene and *Z*-*α*-santalol are shown in Supplementary Fig. 24.

### Reporting summary

Further information on research design is available in the Nature Research Reporting Summary linked to this article.

## Supplementary information

Supplementary Information

Description of Additional Supplementary Files

Supplementary Data 1

Supplementary Data 2

Supplementary Data 3

Supplementary Data 4

Reporting Summary

## Data Availability

The data supporting the findings of this work are available within the paper and its Supplementary Information files. Protein structure data of SaSSy from Protein Data Bank (PDB) was used in this paper for the PDB ID: 5ZZJ. Source data are provided with this paper.

## Source data

Source Data
