# Supplementary material for: Rationally engineering santalene synthase to readjust the component ratio of sandalwood oil
Source: Nat Commun. 2022 May 6;13:2508. doi: 10.1038/s41467-022-30294-8 (PMC9076924; doi:10.1038/s41467-022-30294-8)
Supplement: Supplementary file 1 — Supplementary Information [file 41467_2022_30294_MOESM1_ESM.pdf]

Rationally engineering santalene synthase to readjust the component ratio of sandalwood oil

Zha *et al.*

Supplementary Method 1. Computational details

System setup

The SaSSy and Sansyn models are reconstructed with the intermediates and the Mg^{2+} coordination shell. The AlphaFold2 was used to predict the protein structures (Supplementary Figs. 2 and 25)[1]. The predicted SaSSy structure is highly consistent with its crystal structure in secondary structure (1.25 Å RMSD for structure superposition and 0.984 for TM-score). Thus, the established models were rational for further modeling of SaSSy/SanSyn. The Mg^{2+} coordination shell (including PPi group and three Mg^{2+} ions) was rebuilt based on class I terpene synthases (ATAS and FSTS) with similar conserved coordination motif[2,3]. The protonation states for key residues were also defined by referring to our previous work on ATAS and TEAS[3]. According to the reaction pathways deduced from the products, the (6*S*)-bisabolyl cation intermediate was used as starting point for system setup and subsequent computational studies. The intermediates were docked into SaSSy/SanSyn using Glide (Schrödinger, LLC, New York)[4]. The orientations of the intermediates were preliminarily speculated and selected according to the possible substrate folding modes and relative position between the intermediates and the PPi group[5-7]. Three intermediate conformations of SaSSy models (while only one compact intermediate conformation found for SanSyn) were chosen for further side chain conformation optimization by ABACUS2[8]. The obtained enzyme-ligand complex models were used for further MD simulations to relax the protein structure and intermediate conformation, and the stability of complex structure is validated. The corresponding mutant models were built in the same way as above.

Classical MD simulations

The Amber ff99SB force field[9] was employed for the protein and the TIP3P model was used for water molecules[10]. The force field parameters of the ligands were generated from the general AMBER force field (GAFF)[11], and the partial atomic charge of the substrates was defined by the restrained electrostatic potential (RESP)[12] charge from the HF/6-31G* calculation with the Gaussian 09 package[13]. The initial coordinates and topology files were generated by the *tleap* program in AMBER12[14]. The MD simulations were carried out using the AMBER12 molecular simulation package, and the periodic boundary condition with cubic models were employed. The routine minimization, first by constraining all solute atoms then protein backbone and finally no constraint, were carried out to preliminarily relax the solvent and protein-ligand complex. After the optimization, each system was heated from 0 to 300 K gradually under the NVT ensemble for 100 ps, followed by another 100 ps NPT ensemble MD simulations at 300 K and the target pressure of 1.0 atm. Afterward, 50 ns NVT production MD simulations with a target temperature of 300 K were performed to produce trajectories. During the MD simulations, the SHAKE algorithm[15] was applied to constrain the high-frequency stretching vibration of all hydrogen-containing bonds, and a cutoff of 12 Å was set for vdW (LJ-12 potential) and none cutoff for electrostatic interactions (PME strategy). For SaSSy, two of the three models show similar intermediate conformation after MD simulations (Supplementary Fig. 26), another one

is not stable and turned into non-productive conformation for santalene pathway thus not considered. The intermediate conformation of SanSyn was stable in MD simulations. Snapshots of each system from the stable trajectories were chose to build the initial structures for the subsequent QM/MM simulations.

QM/MM MD simulations

The periodic boundary condition was also considered in the following QM/MM MD simulations. For SaSSy, the T318, F424, S459, F545 and (6*S*)-bisabolyl cation were included in QM region (the charge of QM region is 1). The T298, F404, and F441/V441 together with (6*S*)-bisabolyl cation were included in QM region for wildtype and mutant Sansyn models respectively (the charge of QM region is 0). All of these QM atoms were described with the M06-2X[16,17]/6-31G(d) basis set which is widely used in studying cyclization reaction[18,19], and the model contains about 500 basis functions in total. The QM/MM boundary was treated by the improved pseudo bond approach[20-22]. The same force field in the aforementioned classical MD simulations was used for the remaining atoms. The 12 Å cutoff was employed for both van der Waals interaction by 12-6 Lennard-Jones potential function and electrostatic interactions by dual-focal ai-QM/MM-PME approach[23]. The QM/MM systems were minimized again for several iterations and more than 20 ps QM/MM MD simulations were performed. The resulting conformations of QM/MM MD were used to map out the minimum energy path (MEP) with the reaction coordinate driving (RCD) method[24]. Different RCs were considered for each reaction step and selected based on the obtained MEP. For every point with fixed reaction coordinate, the structure was minimized with microiterative QM/MM. In order to get smooth enough energy profiles, at least three times of forward and backward reaction paths scan were performed. To evaluate the effect of protein dynamics, QM/MM MD umbrella sampling free energy calculations were also performed to compare with the reaction coordinate driven QM/MM scan calculations for the reaction step from B to C state in SaSSy. Each window was calculated for at least 20 ps with 1 fs time steps. The WHAM program was employed to calculate the free energy profile. The convergence of QM/MM MD umbrella sampling can be estimated by the free energy profile gap calculated from different time spans. As a result, the free energy profiles (Supplementary Fig. 27) are highly consist with the relative energy profile (within 1-2 kcal/mol differences). These indicate the protein dynamics may not be significant for the reaction. All of these QM/MM calculations were performed with the interfaced QChem[25]-AMBER12 programs[23].

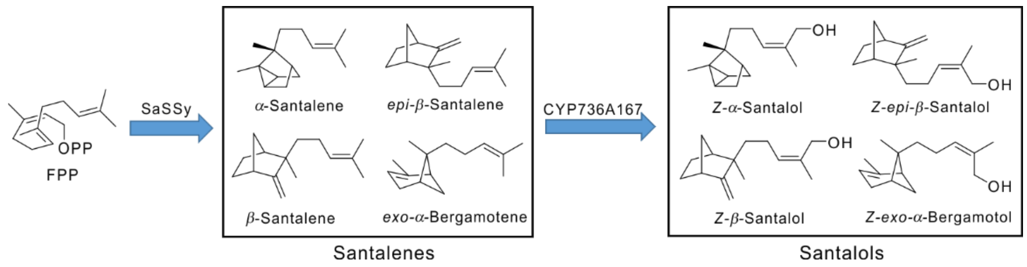

Supplementary Figure 1. The main components of sandalwood oil and their biosynthetic pathway. FPP: farnesyl diphosphate; SaSSy: santalene synthase from *S. album*; CYP736A167: a cytochrome P450 enzyme from *S. album*.

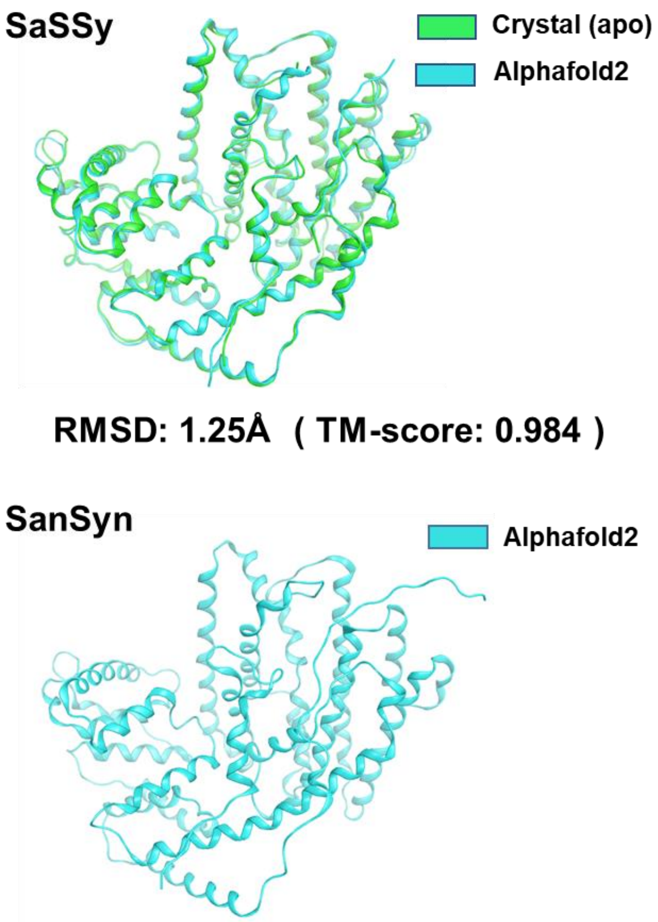

Supplementary Figure 2. Protein models of SaSSy and SanSyn predicted using Alphafold2. The predicted SaSSy is similar with the reported crystal structure of its apo form (TM-score 0.984).

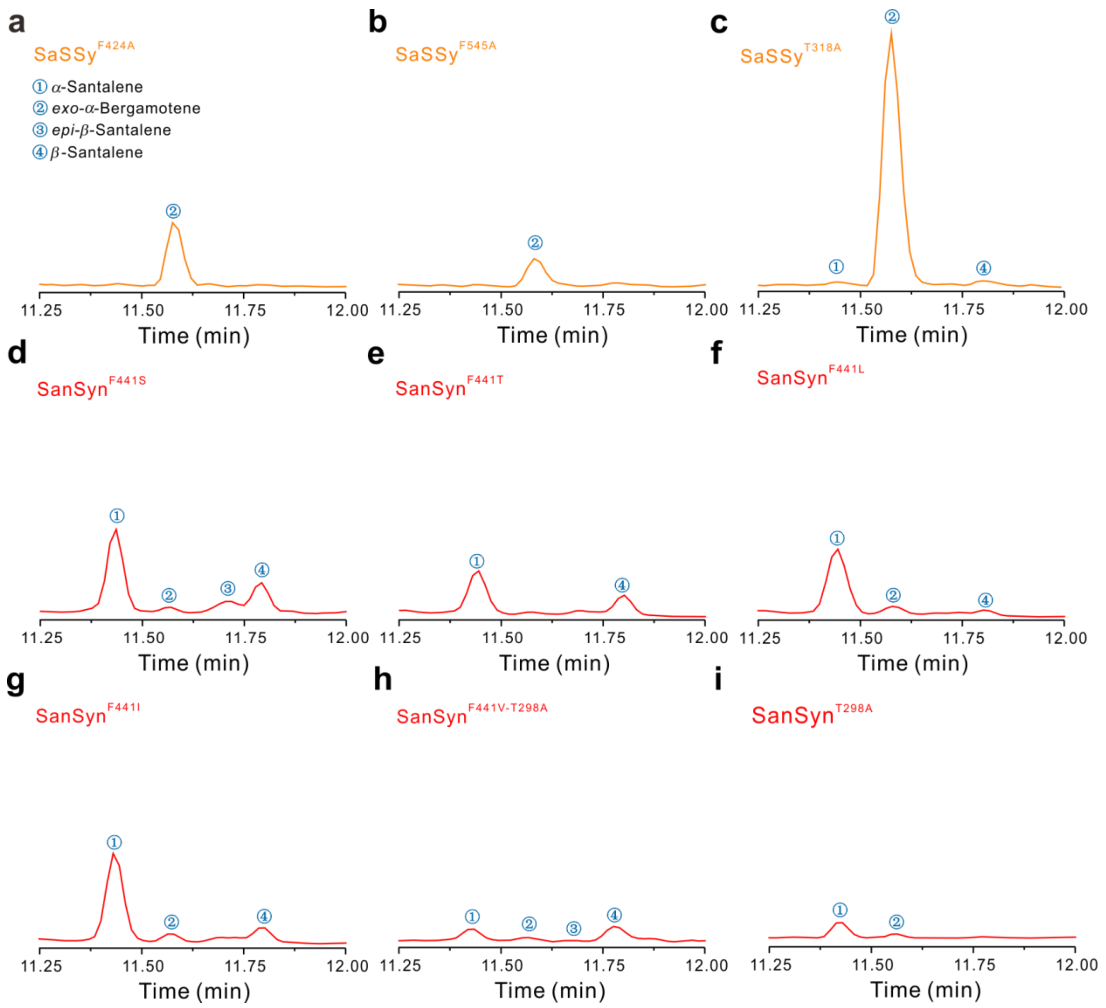

Supplementary Figure 3. GC-MS analysis of the extracts of the yeasts respectively expressing SaSSy^{F424A} (a), SaSSy^{F545A} (b), SaSSy^{T318A} (c), SanSyn^{F441S} (d), SanSyn^{F441T} (e), SanSyn^{F441L} (f), SanSyn^{F441I} (g), SanSyn$^{F441V-T298A}$ (h) and SanSyn^{T298A} (i).

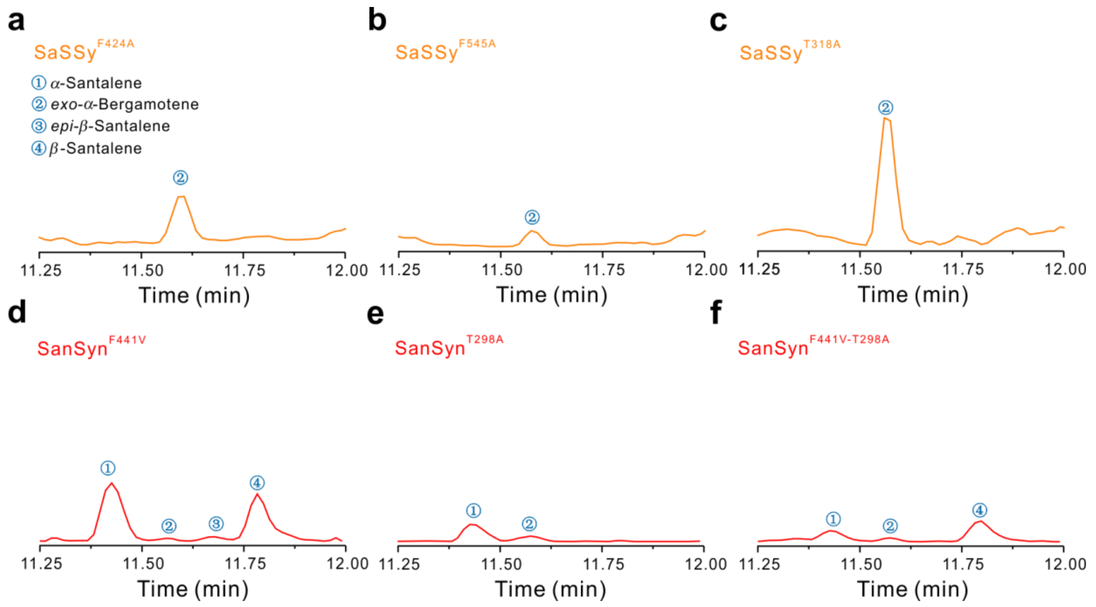

Supplementary Figure 4. GC-MS analysis of *in vitro* reaction mixtures with SaSSy^{F424A} (a), SaSSy^{F545A} (b), SaSSy^{T318A} (c), SanSyn^{F441V} (d), SanSyn^{T298A} (e) and SanSyn$^{F441V\text{-}T298A}$ (f).

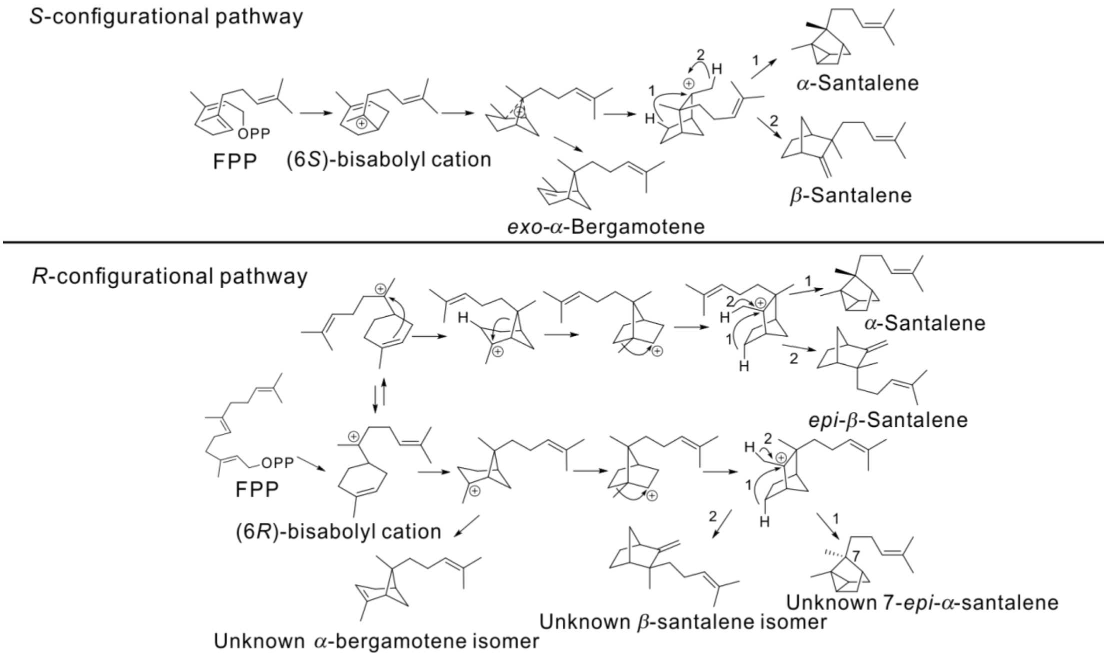

Supplementary Figure 5. The proposed *S*- and *R*-configurational pathways catalyzed by SaSSy.

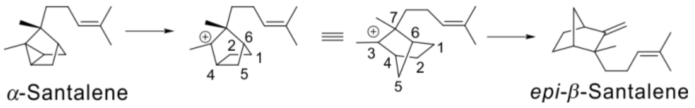

Supplementary Figure 6. Conversion from *α*-santalene to *epi*-*β*-santalene.

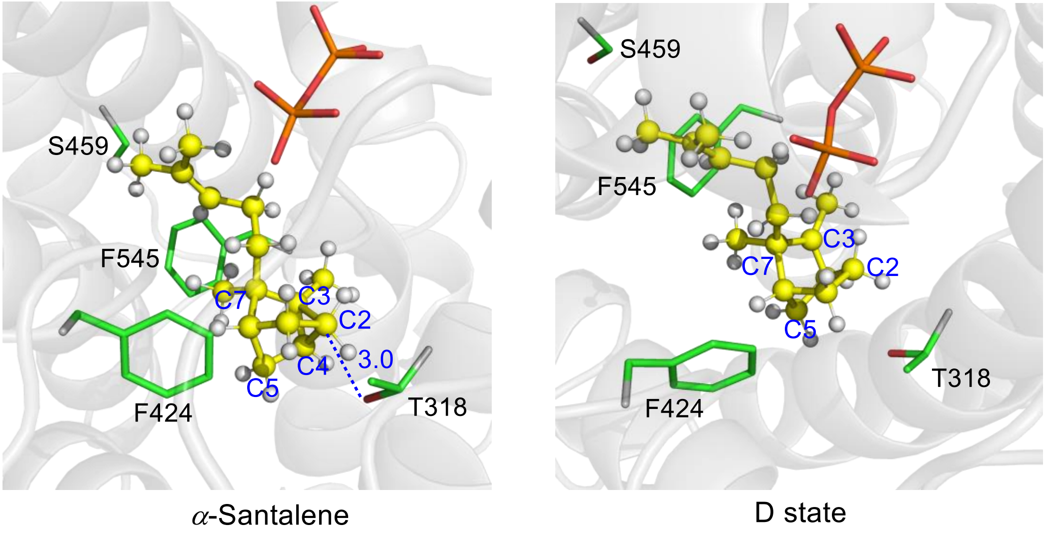

Supplementary Figure 7. The cavity of SaSSy with *α*-santalene or D state.

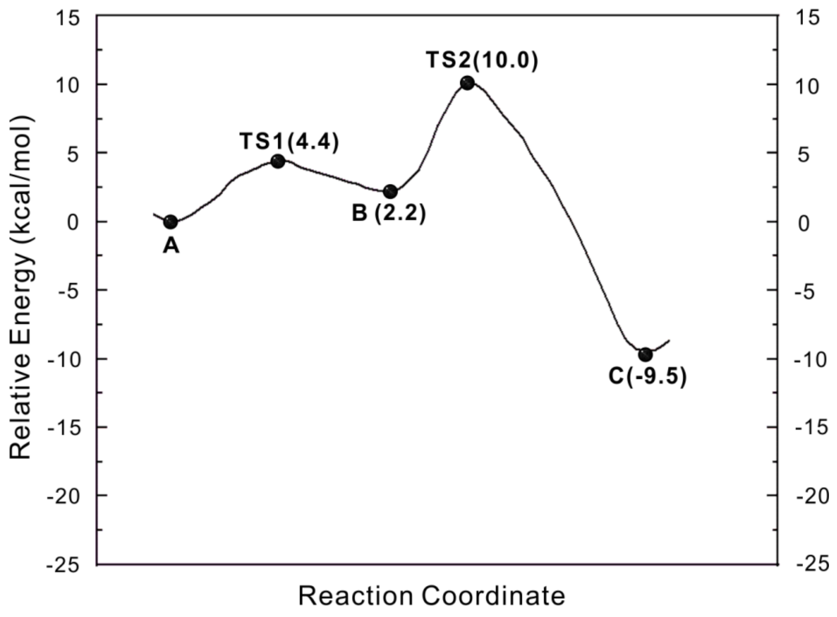

Supplementary Figure 8. QM/MM potential energy profile from A to C states in SanSyn.

```
SaSSy    MDSSTATAMTAPFIDPTDHVNLKTDTDASENRRMGNYKPS    40
SanSyn   ...................MSTQQVSSENIVRNAANFHPN    21

SaSSy    IWNYDFLQSLATHHN.IVEERHLKLAEKLKGQVKFMFGAP    79
SanSyn   IWGNHFLTCPSQTIDSWTQQHHKELKEEVR...KMMVSDA    58

SaSSy    MEPLAKLELVDVVQRLGLNHLFETEIKEALFSIYKDGSNG   119
SanSyn   NKPAQRLRLIDTVQRLGVAYHFEKEIDDALEKIGHDPFDD    98

SaSSy    WWFGHLHATSLRFRLLRQCGLFIPQDVFKTFQNKTGEFDM   159
SanSyn   ..KDDLYIVSLCFRLLRQHGIKISCDVFEKFKDDDGKFKA   136

SaSSy    KLCDNVKGLLSLYEASYLGWKGENILDEAKAFTTKCLKSA   199
SanSyn   SLMNDVQGMLSLYEAAHLAIHGEDILDEAIVFTTTHLKST   176

SaSSy    WEN.ISEKWLAKRVKHALALPLHWRVPRIEARWFIEAYEQ   238
SanSyn   VSNSPVNSTFAEQIRHSLRVPLRKAVPRLESRYFLDIYSR   216

SaSSy    EANMNPTLLKLAKLDFNMVQSIHQKEIGELARWWVTTG.L   277
SanSyn   DDLHDKTLLNFAKLDFNILQAMHQKEASEMTRWWRDFDFL   256

SaSSy    DKLAFARNNLLQSYMWSCA.IASDPKFKLARETIVEIGSV   316
SanSyn   KKLPYIRDRVVELYFWILVGVSYQPKFSTGRIFLSKIICL   296
         T318
SaSSy    LTVVDDGYDVYGSIDELDLYTSSVERWSCVEIDKLPNTLK   356
SanSyn   ETLVDDTFDAYGTFDELAIFTEAVTRWDLGHRDALPEYMK   336
         T298
SaSSy    LIFMSMFNKTNEVGLRVQHERGYNSIPTFIKAWVEQCKSY   396
SanSyn   FIFKTLIDVYSEAEQELAKEGRSYSIHYAIRSFQELVMKY   376

SaSSy    QKEARWFHGGHTPPLEEYSLNGLVSIGFPLLLITGYVAIA   436
SanSyn   FCEAKWLNKGYVPSLDDYKSVSLRSIGFLPIAVASFVFMG   416

SaSSy    EN..EAALDKVHPLPDLLHYSSLLSRLINDIGTSPDEMAR   474
SanSyn   DIATKEVFEWEMNNPKIIIAAETIFRFLDDIAGHRFEQKR   456

SaSSy    GDNLKSIHCYMNETGASEEVAREHIKGVIEENWKILN.QC   513
SanSyn   EHSPSAIECYKNQHGVSEEEAVKALSLEVANSWKDINEEL   496

SaSSy    CFDQSQFQEPFITFNLNSVRGSHFFYEFGDGFGVTDSWTK   553
SanSyn   LLNPMAIPLPLLQVILDLSRSADFMYGNAQDRFTHSTMMK   536

SaSSy    VDMKSVLIDPIPLGEE                           569
SanSyn   DQVDLVLKDPVKLDD.                           551
```

Supplementary Figure 9. Sequence alignment of SanSyn and SaSSy.

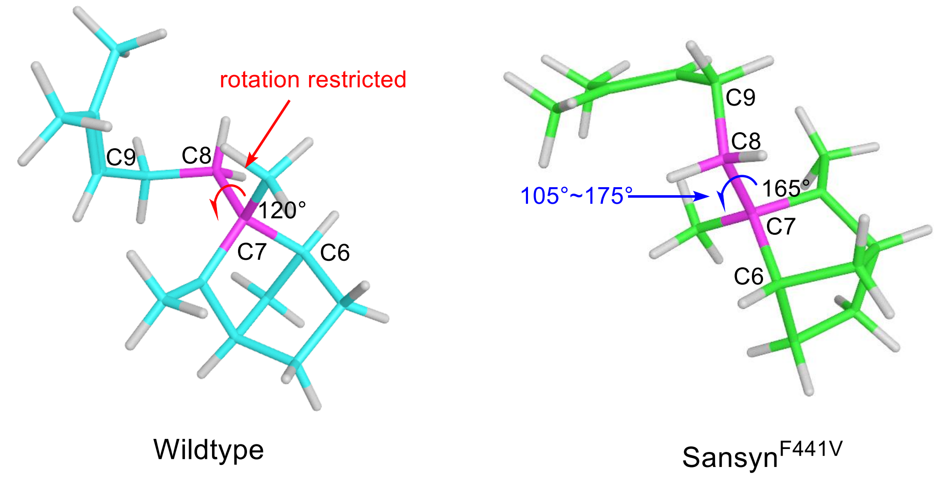

Supplementary Figure 10. The rotatability of C6–C7–C8–C9 dihedral for C state detected in QM/MM MD simulations (20ps) of SanSyn and SanSyn^{F441V}. The rotation at C state is restricted in wildtype, while ranging from 105° to 175° in Sansyn^{F441V}.

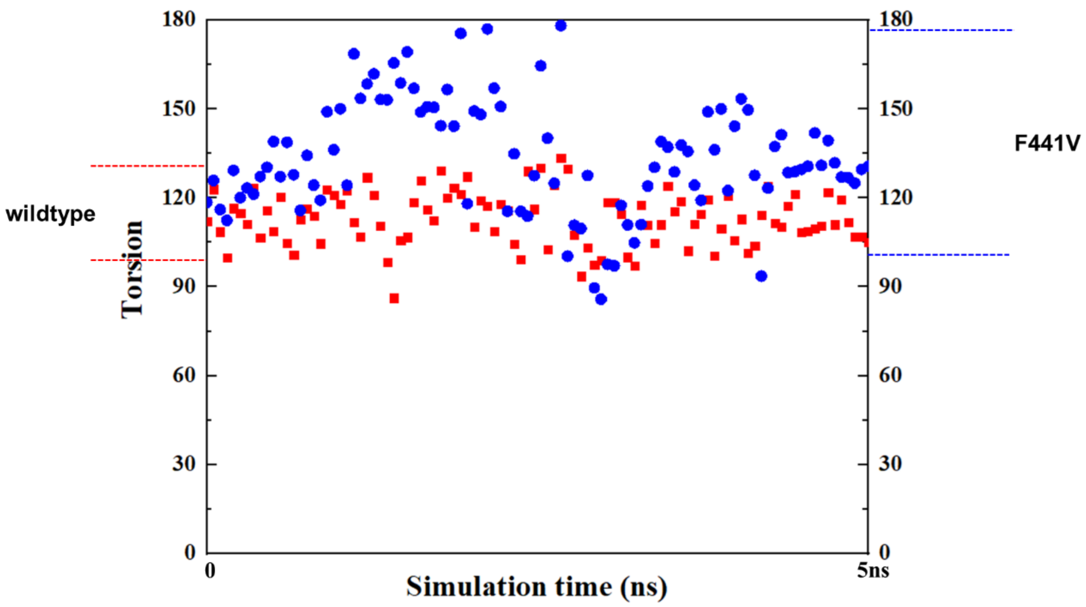

Supplementary Figure 11. The C6–C7–C8–C9 torsion angle of C state in SanSyn wildtype (red) and F441V mutant (blue) during 5ns traditional MD simulations (100 points extracted each, the MD settings were similar as described in computational details).

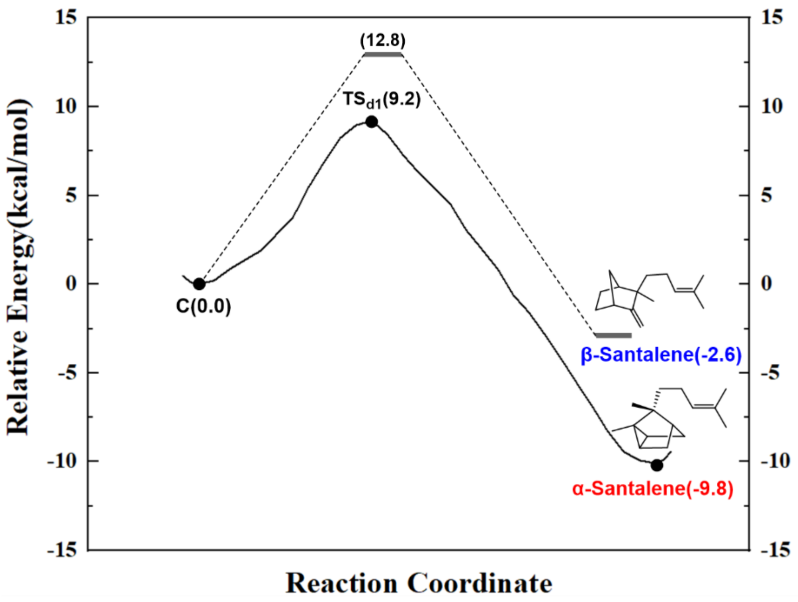

Supplementary Figure 12. The deprotonation from C states to *α* and *β*-santalene with T298 as general base in SanSyn F441V mutant.

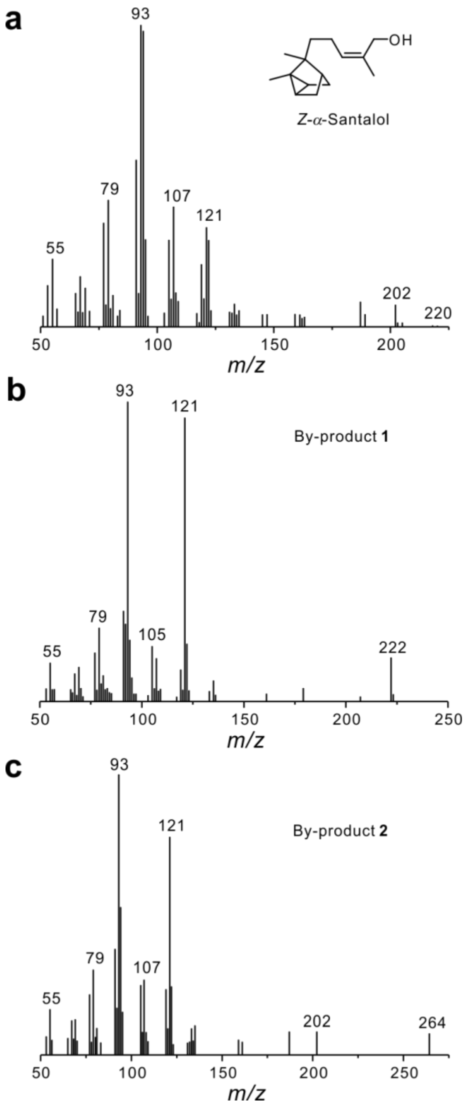

Supplementary Figure 13. The mass spectra of *Z*-*α*-santalol (a), by-product **1** (b) and by-product **2** (c).

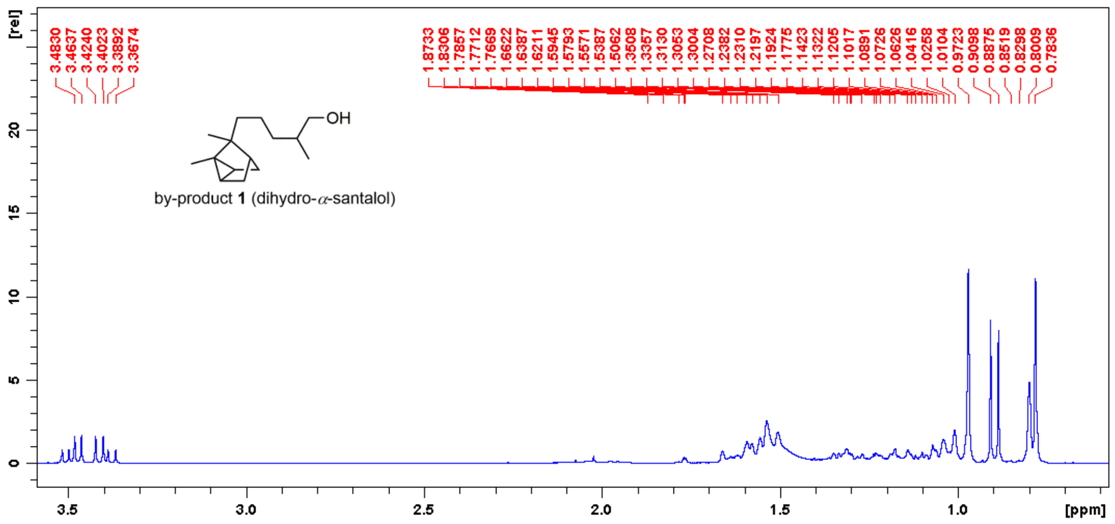

Supplementary Figure 14. ^{1}H NMR (300 MHz, $CDCl_3$) spectrum of by-product **1** (dihydro-*α*-santalol).

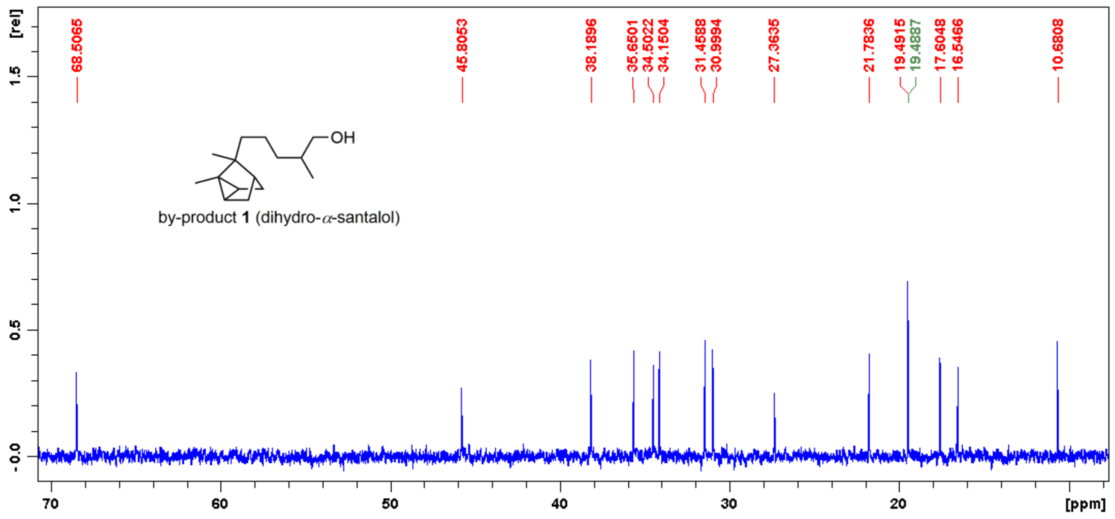

Supplementary Figure 15. ^{13}C NMR (75 MHz, $CDCl_3$) spectrum of by-product **1** (dihydro-*α*-santalol).

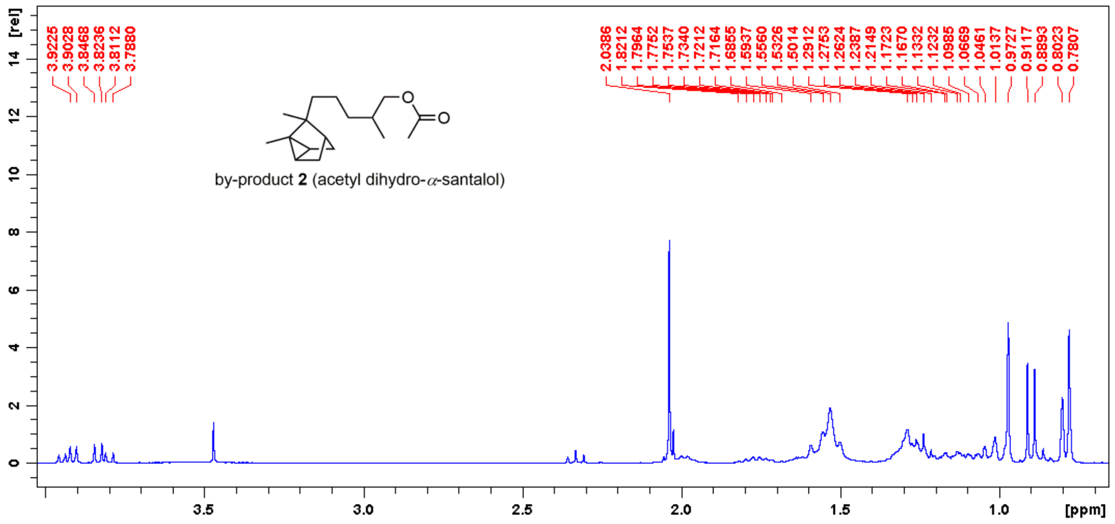

Supplementary Figure 16. ^{1}H NMR (300 MHz, $CDCl_3$) spectrum of by-product **2** (acetyl dihydro-*α*-santalol).

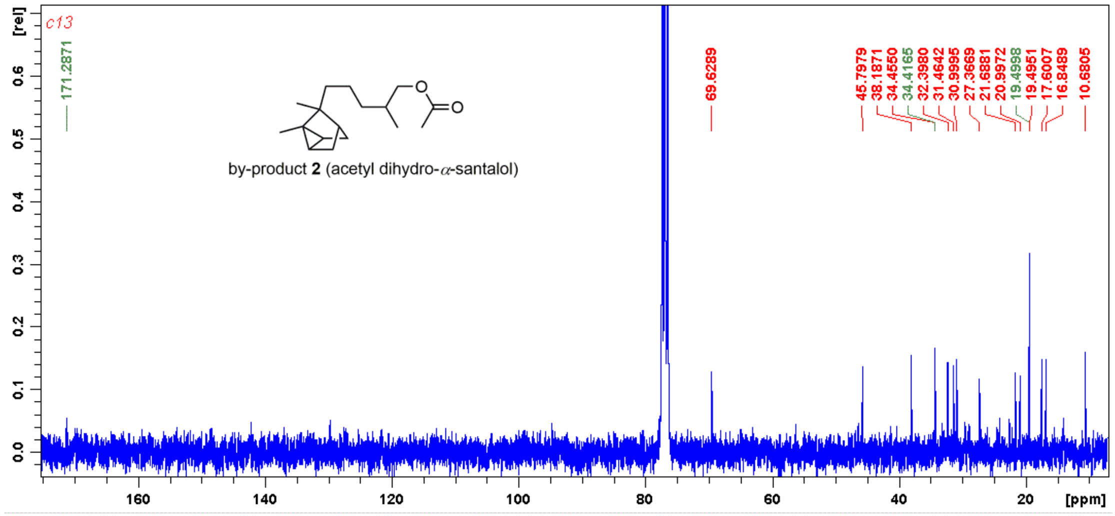

Supplementary Figure 17. ^{13}C NMR (75 MHz, $CDCl_3$) spectrum of by-product **2** (acetyl dihydro-*α*-santalol).

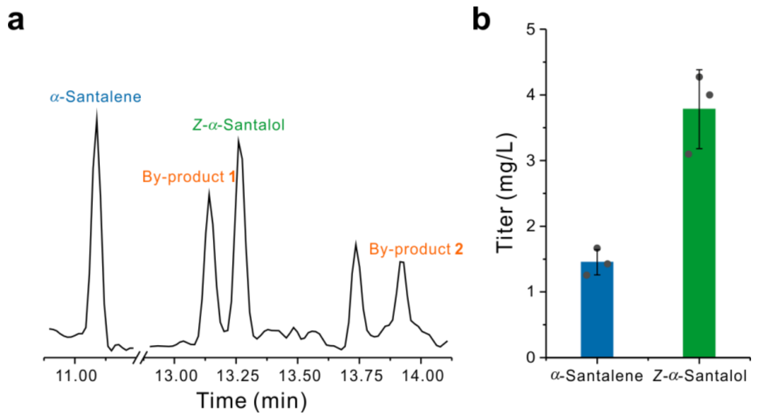

Supplementary Figure 18. Fermentation of SZ12 strain in shake-flasks. (a) GC-MS chromatogram of SZ12 fermentation products. (b) The titers of *α*-santalene and *Z*-*α*-santalol were 1.5 mg/L and 3.8 mg/L, respectively. All data represent the mean of n = 3 biologically independent samples and error bars show standard deviation. Source data are provided as a Source Data file.

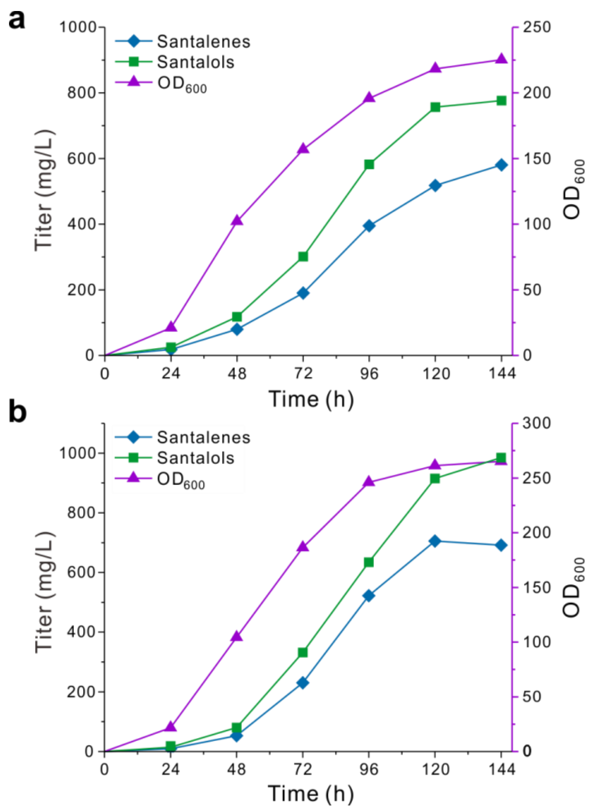

Supplementary Figure 19. The production of SZ22 by fed-batch fermentation. (a) The titers of santalenes and santalols were 580.7 mg/L and 776.4 mg/L when feeding 350 g/L glucose/150 g/L ethanol. (b) The titers of santalenes and santalols were 691.8 mg/L and 984.6 mg/L when feeding 250 g/L glucose/250 g/L ethanol. All data represent the mean of n = 2 biologically independent samples. Source data are provided as a Source Data file.

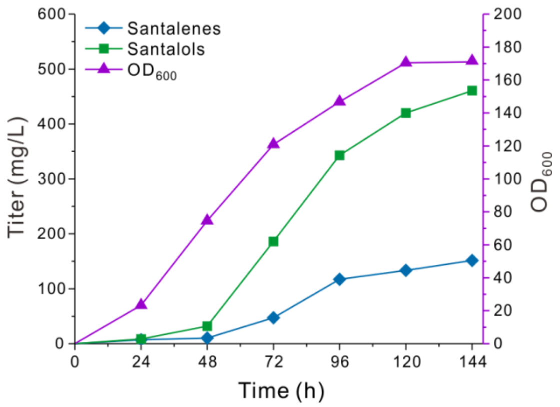

Supplementary Figure 20. Fed-batch fermentation of SZ23 when feeding 150 g/L glucose/350 g/L ethanol. The titers of santalenes and santalols were 151.4 mg/L and 460.6 mg/L, respectively. All data represent the mean of n = 2 biologically independent samples. Source data are provided as a Source Data file.

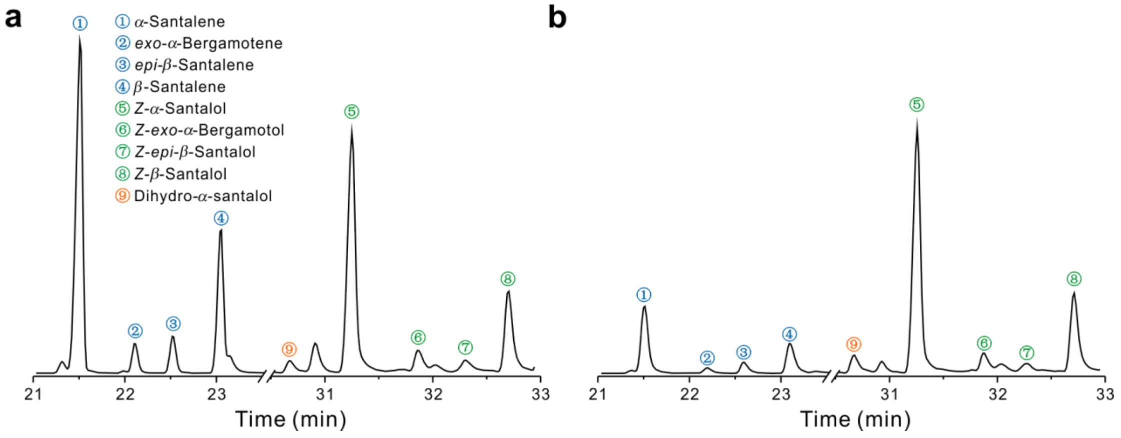

Supplementary Figure 21. GC-MS chromatograms of SZ22 (a) and SZ24 (b) fermentation products in fed-batch fermentation when feeding 150 g/L glucose/350 g/L ethanol.

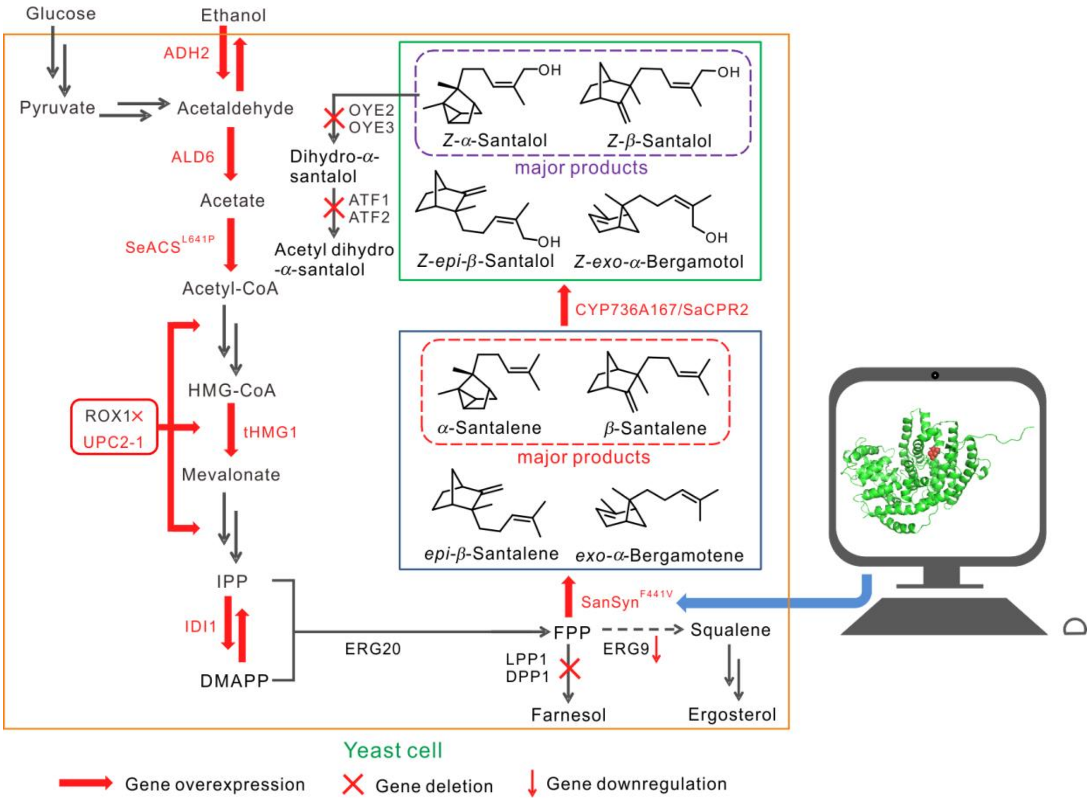

Supplementary Figure 22. Biosynthetic pathway for santalenes and santalols in engineered yeasts. Acetyl-CoA, Acetyl coenzyme A; HMG-CoA, 3-hydroxy-3-methylglutaryl-CoA; IPP, isopentenyl pyrophosphate; DMAPP, dimethylallyl pyrophosphate; FPP, farnesyl diphosphate; LPP1 and DPP1, phosphate phosphatases; OYE2 and OYE3, old yellow enzymes; ROX1, a yeast transcriptional repressor; ATF1 and ATF2, alcohol acetyltransferases; ADH2, yeast alcohol dehydrogenase; ALD6, yeast acetaldehyde dehydrogenase; SeACS^{L641P}, *Salmonella enterica* acetyl-CoA synthetase L641P variant; UPC2-1, a mutant of the transcription factor UPC2; tHMG1, the truncated 3-hydroxy-3-methylglutaryl coenzyme A reductase; IDI1, isopentenyl diphosphate isomerase; ERG20, yeast farnesyl pyrophosphate synthetase; ERG9, yeast squalene synthase; SanSyn^{F441V}, a mutant of santalene synthase from *C. lansium*; CYP736A167, a cytochrome P450 enzyme from *S. album*; SaCPR2, a cytochrome P450 reductase from *S. album*.

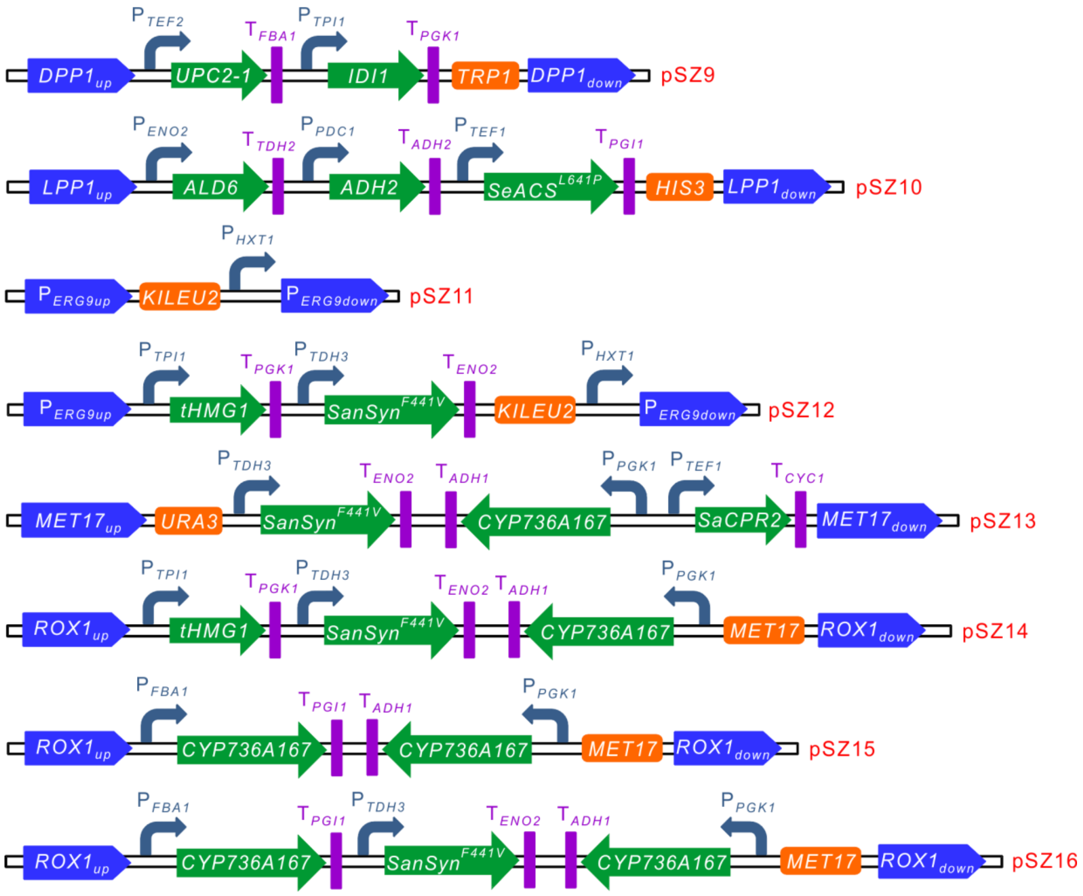

Supplementary Figure 23. Pathway engineering strategy for strains construction, all expression modules were connected with pCFB2988 vector.

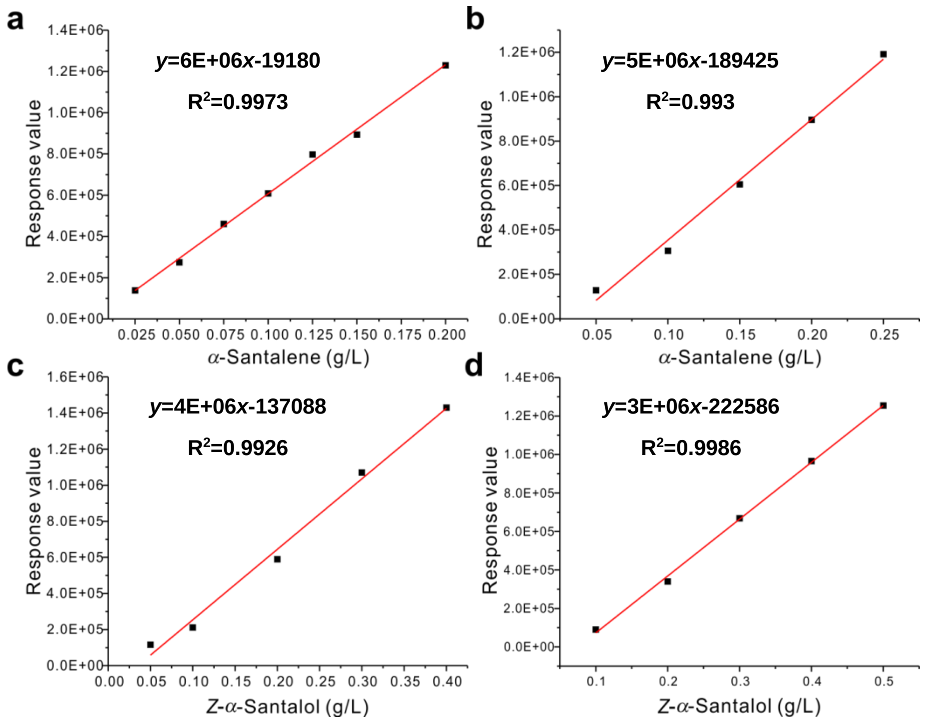

Supplementary Figure 24. The external calibration curves of *α*-santalene and *Z*-*α*-santalol. (a) and (c) for quantification of SZ1-SZ20 strains, were blotted under the condition: the temperature gradient program started from 50 °C, and after a 3 min hold, raised by 20 °C/min to 70 °C, then raised by 15 °C/min to 300 °C where it was hold for 3 min. (b) and (d) for quantification of SZ21-SZ24 strains, were blotted under the condition: the temperature gradient program also started from 50 °C, and after a 3 min hold, raised to 70 °C by 20 °C/min and hold 1 min, then to 160 °C by 3 °C/min, and finally to 300 °C by 20 °C/min.

PROCHECK results of SaSSy model

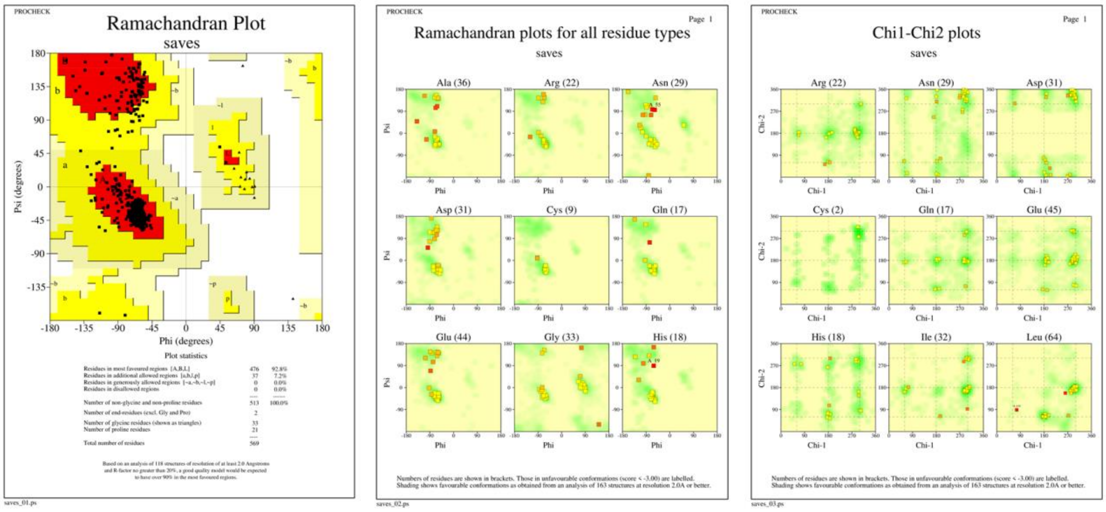

PROCHECK results of Sansyn model

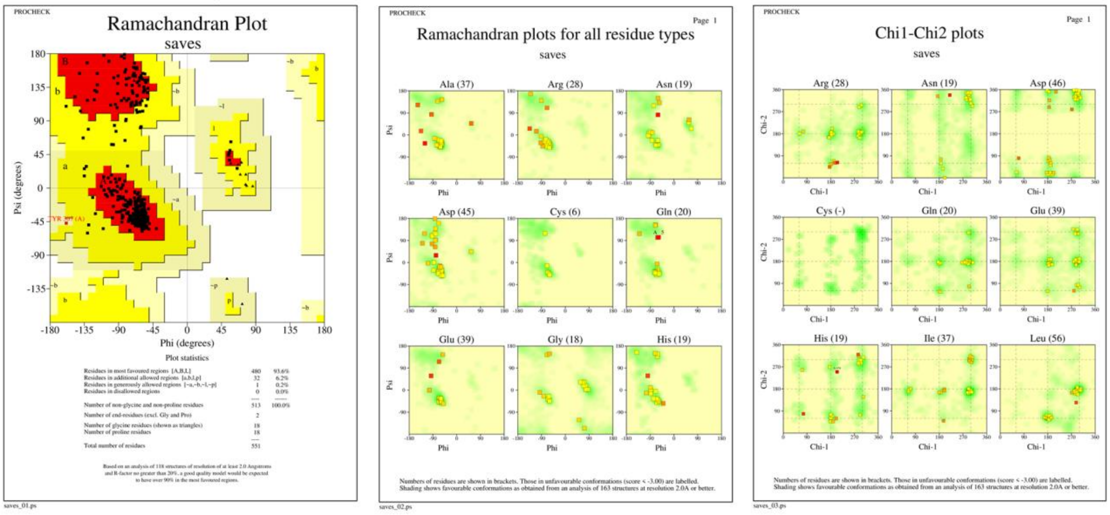

Supplementary Figure 25. The PROCHECK results of SaSSy and Sansyn models indicate the good quality of the models predicted using Alphafold2.

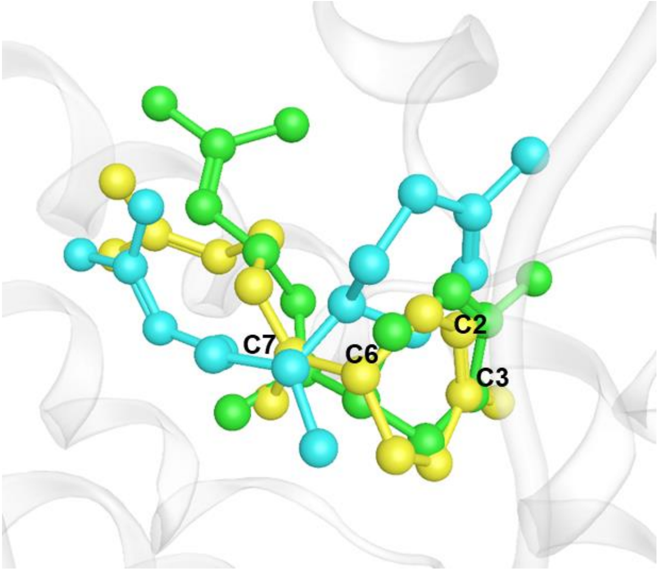

Supplementary Figure 26. The intermediate conformations in SaSSy models after MD simulations. The yellow and green ones show similar preorgnized conformation for santalene pathway, while the cyan one is not productive conformation for santalene pathway.

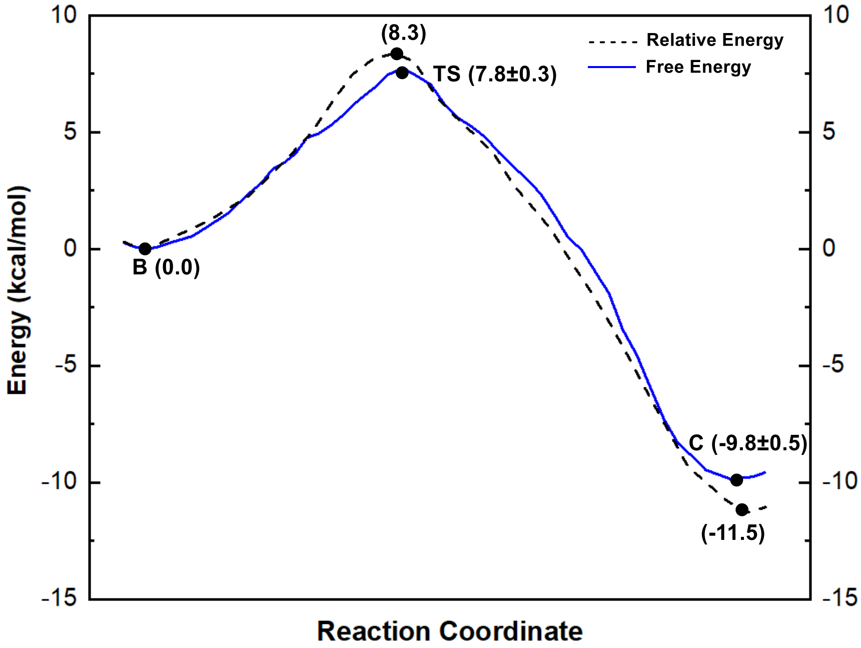

Supplementary Figure 27. The relative energy (dashed black) and free energy (blue) profiles from B to C state in SaSSy.

Supplementary references

1. Tunyasuvunakool, K. et al. Highly accurate protein structure prediction for the human proteome. *Nature* **596**, 590−596 (2021).
2. Chen, M. et al. Mechanistic insights from the binding of substrate and carbocation intermediate analogues to aristolochene synthase. *Biochemistry* **52**, 5441 (2013).
3. Zhang, F. et al. Enzyme promiscuity versus fidelity in two sesquiterpene cyclases (TEAS versus ATAS). *ACS Catal*. **10**, 1470–1484 (2020).
4. Friesner, R. A. et al. Extra precision Glide: docking and scoring incorporating a model of hydrophobic enclosure for protein−ligand complexes. *Med. Chem*. **49**, 6177 (2006).
5. O'Brien, T. E., Bertolani, S. J., Tantillo, D. J. & Siegel, J. B. Mechanistically informed predictions of binding modes for carbocation intermediates of a sesquiterpene synthase reaction. *Chem. Sci*. **7**, 4009 (2016).
6. O'Brien, T. E., Bertolani, S. J., Zhang, Y., Siegel, J. B. & Tantillo, D. J. Predicting productive binding modes for substrates and carbocation intermediates in terpene synthases—bornyl diphosphate synthase as a representative case. *ACS Catal*. **8**, 3322 (2018).
7. Wang, Y. H., Xie, H., Zhou, J., Zhang, F. & Wu, R. Substrate folding modes in trichodiene synthase: a determinant of chemo- and stereoselectivity. *ACS Catal*. **7**, 5841 (2017).
8. Xiong, P. et al. Increasing the efficiency and accuracy of the ABACUS protein sequence design method. *Bioinformatics* (2019).
9. Duan, Y. et al. A Point-charge force field for molecular mechanics simulations of proteins based on condensed-phase quantum mechanical calculations. *Comput. Chem*. **24**, 1999 (2003).
10. Jorgensen, W. L., Chandrasekhar, J., Madura, J. D., Impey, R. W. & Klein, M. L. Comparison of simple potential functions for simulating liquid water. *J. Chem. Phys*. **79**, 926 (1983).
11. Wang, J., Wolf, R. M., Caldwell, J. W., Kollman, P. A. & Case, D. A. Development and testing of a general AMBER force field. *J. Comput. Chem*. **25**, 1157 (2004).
12. Bayly, C. I., Cieplak, P., Cornell, W. & Kollman, P. A. A well-behaved electrostatic potential based method using charge restraints for deriving atomic charges: the RESP model. *J. Phys. Chem*. **97**, 10269 (1993).
13. Frisch, M. J. T. et al. Gaussian 09, *Gaussian Inc., Wallingford, CT* (2009).
14. Case, D. A. et al. AMBER12, *University of California, San Francisco, CA* (2012).
15. Ryckaert, J. P. C., Ciccotti, G. & Berendsen, H. J. C. Numerical integration of the cartesian equations of motion of a system with constraints: molecular dynamics

of n-alkanes. *J. Comput. Phys*. **23**, 327 (1977).

16. Zhao, Y. & Truhlar, D. G. Construction of a generalized gradient approximation by restoring the density-gradient expansion and enforcing a tight lieb–oxford bound. *Theor. Chem. Acc.* **120**, 215 (2008).
17. Zhao, Y. & Truhlar, D. G. Exploring the limit of accuracy of the global hybrid meta density functional for main-group thermochemistry, kinetics, and noncovalent interactions. *J. Chem. Theory Comput*. **4**, 1849 (2008).
18. Chen, N., Zhou, J., Li, J., Xu, J. & Wu, R. Concerted cyclization of lanosterol c-ring and d-ring under human oxidosqualene cyclase catalysis: an ab initio QM/MM MD study. *J. Chem. Theory Comput.* **10**, 1109 (2014).
19. Chen, N., Wang, S., Smentek, L., Hess, B. A., Jr. & Wu, R. Biosynthetic mechanism of lanosterol: cyclization. *Angew. Chem. Int. Ed.* **54**, 8693 (2015).
20. Chu, Y., Xu, Q. & Guo, H. Understanding energetic origins of product specificity of SET8 from QM/MM free energy simulations: what causes the stop of methyl addition during histone lysine methylation?. *J. Chem. Theory Comput.* **6**, 1380 (2010).
21. Chu, Y., Yao, J. & Guo, H. QM/MM MD and free energy simulations of G9a-like protein (GLP) and its mutants: understanding the factors that determine the product specificity. *Plos One* **7**, e37674 (2012).
22. Hu, P., Wang, S. & Zhang, Y. How do set-domain protein lysine methyltransferases achieve the methylation state specificity? revisited by ab initio QM/MM molecular dynamics simulations. *J. Am. Chem. Soc.* **130**, 3806 (2008).
23. Zhou, Y., Wang, S., Li, Y. & Zhang, Y. Born–oppenheimer ab initio QM/MM molecular dynamics simulations of enzyme reactions. *Methods Enzymol*. **577**, 105 (2016).
24. Zhang, Y., Liu, H. & Yang, W. Free energy calculation on enzyme reactions with an efficient iterative procedure to determine minimum energy paths on a combined ab initio QM/MM potential energy surface. *J. Chem. Phys*. **112**, 3483 (2000).
25. Shao, Y. et al. Advances in methods and algorithms in a modern quantum chemistry program package. *Phys. Chem. Chem. Phys.* **8**, 3172 (2006).
