# Supplementary material for: Rationally engineering santalene synthase to readjust the component ratio of sandalwood oil
Source: Nat Commun. 2022 May 6;13:2508. doi: 10.1038/s41467-022-30294-8 (PMC9076924; doi:10.1038/s41467-022-30294-8)
Supplement: Supplementary file 2 — Description of Additional Supplementary Files [file 41467_2022_30294_MOESM2_ESM.pdf]

Description of Additional Supplementary Files

File Name: Supplementary Data 1
Description: List of strains used in this study.

File Name: Supplementary Data 2
Description: List of plasmids used in this work.

File Name: Supplementary Data 3
Description: List of primers used in this work.

File Name: Supplementary Data 4
Description: PDB files of SaSSy and SanSyn computational models.
